# A Novel Lipopolysaccharide Recognition Mechanism Mediated by Internalization in Teleost Macrophages

**DOI:** 10.3389/fimmu.2018.02758

**Published:** 2018-11-27

**Authors:** Xin-Jiang Lu, Ying-Jun Ning, He Liu, Li Nie, Jiong Chen

**Affiliations:** ^1^Laboratory of Biochemistry and Molecular Biology, Ningbo University, Ningbo, China; ^2^Key Laboratory of Applied Marine Biotechnology of Ministry of Education, Ningbo University, Ningbo, China

**Keywords:** teleost, macrophages, lipopolysaccharide, scavenger receptor class B2, inflammation

## Abstract

Macrophages in teleosts are less sensitive to lipopolysaccharide (LPS) compared to mammals. The functional equivalent of the mammalian LPS surface receptor in teleost macrophages for the pro-inflammatory response is either non-existent or replaced by negative regulation. LPS signaling in teleost macrophages remains unclear. Here, we found a scavenger receptor class B 2a (PaSRB2a) that played a crucial role in LPS signaling in teleost macrophages. The internalization of LPS and subsequent pro-inflammatory responses in macrophages were mediated by PaSRB2a, which is a novel isoform of the mammalian SRB2 gene. LPS internalization by PaSRB2a is dependent on its C-terminal intracellular domain. Following LPS internalization, it interacts with the ayu intracellular receptors nucleotide-binding oligomerization domain protein 1 (PaNOD1) and PaNOD2. Moreover, LPS pre-stimulation with sub-threshold concentrations reduced the effect of secondary LPS treatment on pro-inflammatory responses that were mediated by PaSRB2a. The pro-inflammatory responses in LPS-treated ayu were down-regulated upon PaSRB2a knockdown by lentivirus siRNA delivery. In grass carp and spotted green pufferfish, SRB2a also mediated LPS internalization and pro-inflammatory responses. Our work identifies a novel LPS signaling pathway in teleosts that differs from those in mammals, and contributes to our understanding of the evolution of pathogen recognition in vertebrates.

## Introduction

Lipopolysaccharides (LPS), a major component of the outer membrane of gram-negative bacteria, are sensed by immune receptors (1). Since LPS commonly exists in pathogenic gram-negative bacteria, it has been identified as a major endotoxin in infectious diseases in vertebrates (2, 3). Macrophages are the main target cells of LPS (4, 5). After LPS recognition, macrophages are activated to produce pro-inflammatory cytokines (6). It has long been known that leukocytes of teleosts are less sensitive to LPS compared with those of mammals (7–9). This suggests that the mechanisms of LPS recognition differ between teleosts and mammals.

Extracellular LPS recognition plays an important role in pro-inflammatory responses (10, 11). Toll-like receptors (TLRs) participate in innate immune recognition as pattern recognition receptors (12–14). Among the TLRs, TLR4 was the first to be identified and is involved in the recognition of LPS, which is implicated in the etiology of LPS-induced septic shock (15). The TLR4 signaling pathway mediated by extracellular LPS recognition also includes myeloid differentiation protein 2 (MD2) and CD14 (16). In TLR4 knockout mouse macrophages, even 100 μg/ml LPS does not induce up-regulation of TNF, suggesting the critical role of TLR4 in LPS recognition of mammalian macrophages (17). Moreover, human TLR2 is also identified as a LPS receptor (18). In teleosts, TLR4 is lacking in some species and has been identified as a negative regulator of TLR signaling in zebrafish (19). The CD14 and MD2 genes of the TLR4 signaling pathway are not found in teleost genomes (19, 20). TLR2 genes have been identified in several teleosts species (zebrafish, Japanese flounder, fugu, and catfish). The precise mechanisms underlying extracellular LPS recognition in teleosts are unclear.

In addition to surface receptor interaction, LPS can also activate pro-inflammatory responses by binding with intracellular receptors via internalization by electroporation (21–23). In the inflammasome, intracellular sensor protein activation regulates the down-stream nuclear factor-κB (NF-κB), which participates in the pro-inflammatory response (24, 25). Nucleotide-binding oligomerization domain protein 1 (NOD1) has been implicated in the recognition of intracellular bacteria and LPS not only in mammals (26), but also in teleosts (27, 28), suggesting that the intracellular LPS recognition pattern is similar in mammals and teleosts. In mammals, outer membrane vesicles of bacteria are internalized by macrophages and release LPS in the cytoplasm (23, 29). It is still unclear how LPS enters the cytoplasm of macrophages in teleosts.

Teleost macrophage studies have revealed notable similarities and differences in the molecular strategies by which fish and higher vertebrates control their pro-inflammatory responses (6, 30). Here, we have found a novel pathway for LPS entrance into the cytoplasm of the teleost macrophages, via binding to a scavenger receptor. The ayu scavenger receptor B 2a (PaSRB2a) mediated the pro-inflammatory response in macrophages by interacting with LPS and internalizing it, activating NOD1 and NOD2. SRB2a expression was further confirmed to be dramatically down-regulated after LPS treatment in ayu macrophages, which partially explains the LPS tolerance of teleosts. Furthermore, SRB2a, a novel gene which is common in teleosts but not in mammals, was also shown to play an important role in LPS internalization and pro-inflammatory responses in grass carp and spotted green pufferfish.

## Materials and methods

### Animals

Ayu (*Plecoglossus altivelis*), weighing 40 ± 5 g each, were used in all experiments. This strain has undergone seven successive generations of mass selection (31). The fish were kept at 20–22°C in a recirculating system using filtered water. They were held in the laboratory for two weeks before use in experiments. Grass carp, with a body weight of 100−120 g, were obtained from a commercial farm (Hunan, China). The fish were kept in freshwater tanks at 20–22°C in a recirculating system. One-year old wild-type spotted green pufferfish, weighing 3–6 g, were kept in recirculating water at 26–28°C. They were held in the laboratory for at least 2 weeks, with healthy appearance and normal activity, prior to use in experiments. For cell cultures, 7–8 fish were used in each experiment. The experimental conditions and procedures were approved by the Ningbo University Institutional Animal Care and Use Committee and were carried out in compliance with the National Institutes of Health's Guide for the Care and Use of Laboratory Animals.

### Lipopolysaccharide extraction and quantification

LPS was extracted from *Aeromonas hydrophila* (ATCC 7966) and *Vibrio anguillarum* (Ayu-H080701) with the Lipopolysaccharide Extraction Kit (iNtRON Biotechnology, Inc., Burlington, MA, USA) according to the manufacturer's instructions. LPS extract was dissolved and treated with 2 mg/ml proteinase K at 37°C overnight, then collected by ethanol precipitation. After washing with 70% ethanol, pellets were dried, weighed, and reconstituted at a concentration of 1 mg/ml in distilled water. LPS purity was confirmed by its inability to induce IL-6 production in macrophages isolated from the LPS-unresponsive C3H/HeJ mouse strain (32). LPS from *Escherichia coli* O55:B5 and O111:B4 was purchased from Sigma Aldrich (St. Louis, MO, USA). Purpald assay was employed to quantify LPS. To each of the duplicated wells, we added 50 μl of the sample and 50 μl of 32 mm NaIO_4_ before incubating at room temperature for 25 min in the dark. After this, purpald reagent was added and the plate was incubated for 20 min in the dark. After NaIO_4_ was added, the absorbance was read at 550 nm.

### Characterization of gene cDNAs

The cDNA sequence of the PaSRB2a and PaSRB2b genes were obtained by the transcriptome analysis of ayu macrophages, and transcriptomic data were deposited in the Gene Expression Omnibus (http://www.ncbi.nlm.nih.gov/geo/) under accession number GSE40221 (33). PCR, cloning, and sequencing were used to confirm the authenticity of these sequences. The partial cDNA sequence of PaTLR2a, PaNOD1, and PaNOD2 genes were also obtained by transcriptome analysis of ayu macrophages. The full-length cDNA sequence of PaTLR2a, PaNOD1, and PaNOD2 were subsequently obtained by using the rapid amplification of cDNA ends (RACE) method and the sequence specific primers. First strand cDNA was synthesized using the SMART RACE kit (Clontech, Palo Alto, CA, USA). Gene-specific primers for PaNOD1, PaNOD2, and PaTLR2a were designed based on the corresponding gene sequences (Table 1). The cDNA sequences of PaTLR2a, PaSRB2a, PaSRB2b, PaNOD1, and PaNOD2 were deposited in GenBank under accession numbers MG674831, MH699855, JP736791, MG674829, and MG674830, respectively.

**Table 1:** Oligonucleotide primers used for amplifying cDNAs, expressing constructs, and gene expression analysis.

**Primer**	**Accession number**	**Nucleotide sequence (5'-3')**	**Use**
PaNOD1pF	MG674829	GC*GAATTC*ATGCACGTGAACATGAACGAG^a^	Prokaryotic expression
PaNOD1pR		GC*CTCGAG*TCATCCTGTTCCTTGAAGTGC^a^
PaNOD2pF	MG674830	GC*GAATTC*ATGAGTGCCCAGCAGTTGGT^a^	Prokaryotic expression
PaNOD2pR		GC*CTCGAG*TCATGTTTGAGTTCATCCAGG^a^
PaSRB2aF	MH699855	GGCGCCGGCGCAATGACCCGAAGATCCTGTGa^a^	Eukaryotic expression
PaSRB2aR		GC*GCGATCGC*TCAAATCTCGCTTTGTGCGA^a^
PaSRB2bF	JP736791	GG*CGCCGGCG*CAATGTCAATGAAATATTGCTGC^a^	Eukaryotic expression
PaSRB2bR		GC*GCGATCGC*CTACGATGAAGCCAACAGG^a^
PaNOD1F	MG674829	GG*CGCCGGCG*CAATGCACGTGAACATGAACGAG^a^	Eukaryotic expression
PaNOD1R		GC*GCGATCGC*GTGGAAGCGAAGCCTTGGC^a^
PaNOD2F	MG674830	G*CGGCGCCG*GCGCAATGAGTGCCCAGCAGTTGGTG^a^	Eukaryotic expression
PaNOD2R		GC*GCGATCGC*TCACTGATTTACATACCACACTG^a^
PaTLR2aF	MG674831	GG*CGCCGGCG*CAGGGGTGAGGGCTGAGGGT^a^	Eukaryotic expression
PaTLR2aR		GC*GCGATCGC*TCAGTCGTCCCCGTTCAGAGC^a^
PaNOD1F1	MG674829	TCCCGGAAAGTCTTGACCGC	3'RACE
PaNOD1R1		ATTGATGAGCAGCAGCAGAGG	5'RACE
PaNOD2R1	MG674830	GTTATTTCCCAGCCTGAGGGAG	5'RACE
PaTLR2F1	MG674831	GCATGGAGGCCAGGACGTT	3'RACE
PaTLR2R1		AACGACCTGAGGAGCCTTGAC	5'RACE
PaNOD1F	MG674829	CAAGATTGGCAGCAACAAGA	RT-qPCR
PaNOD1R		GAGAGGCTCAGGTTGGTCAG
PaNOD2F	MG674830	TGCCCTCTTCAACAACAAGC	RT-qPCR
PaNOD2R		TGCTCCAACATCCCCAATCT
PaSRB2aF	MH699855	ACTTCTACCAAGCAGACCCC	RT-qPCR
PaSRB2aR		GGGGAAGATGGTCTGGTTGA
PaSRB2bF	JP736791	ATGTTCTCGTCGGACCTCTG	RT-qPCR
PaSRB2bR		GCATCGGCCTGGTAGAAATG
PaTLR2aF	MG674831	TTGGACAGGCTCACACATCT	RT-qPCR
PaTLR2aR		AAGCAGTTGTTTCAGCCTGG
PaTLR2bF	GFIR01037304	TGGTGGTAAAAGAGCCCATC	RT-qPCR
PaTLR2bR		TCAACTGCGGAGACCTTTCT
IL-1βF	HF543937	TACCGGTTGGTACATCAGCA	RT-qPCR
IL-1βR		TGACGGTAAAGTTGGTGCAA
TNF-αF	JP740414	ACATGGGAGCTGTGTTCCTC	RT-qPCR
TNF-αR		GCAAACACACCGAAAAAGGT
CD115F	KT692936	TGTACACCGTCCAGAGTGAC	RT-qPCR
CD115R		AATTGTTCGGAAAGTGGGCC
18SF	FN646593	GAATGTCTGCCCTATCAACT	RT-qPCR
18SR		GATGTGGTAGCCGTTTCT
PaSRB2a-siRNA-sense	MH699855	GATCCCCCCTTGACCTTAATCCGACCACTGGTTTCAAGAGAACCAGTGGTCGGATTAAGGTCAAGGTTTTTA	Lentiviral RNAi
PaSRB2a-siRNA-antisense		AGCTTAAAAACCTTGACCTTAATCCGACCACTGGTTCTCTTGAAACCAGTGGTCGGATTAAGGTCAAGGGGG
PaNOD1-siRNA	MG674829	GAGCUACAGACAGACGCCAUGUUCU	Stealth RNAi
PaNOD2-siRNA	MG674830	CAGCUUUACGGUGUGUGAUUAUGAU
PaSRB2a-siRNA	MH699855	CCGGGAUAGUUUGCGCUCAUCUACU
CiSRB2a-siRNA	MH699856	CAGCAGAGUAUUGGAGGGCUGGAUA
TnSRB2a-siRNA	ENSTNIT00000018885	GCUGUUCAACAAAUCGGGCCAUAUA
PaSRB2b-siRNA	JP736791	CACACACUGUUGGAGAGCUGCUUUG
PaTLR2-siRNA	MG674831	GACCUCACGUUAGCUGAGACGCUUU
Scrambled-siRNA		CACGUCAGGUUGAGACGUCUCAUUG

a *The underlined nucleotides indicate the restriction sites for restriction endonucleases*.

The similarity between the obtained sequences and other known sequences were analyzed using the basic local alignment search tool (https://blast.ncbi.nlm.nih.gov/). Phylogenetic and molecular evolutionary analyses were conducted using the molecular evolutionary genetics analysis (MEGA) tool (version 5). The accession numbers of TLR genes are listed in Table 2. The accession numbers of SRB genes are listed Table 3.

**Table 2:** TLR1-4 sequences used in this study.

**Accession no. ensemble ID**	**Species**		**Protein**

	**Latin name**	**English name**
FJ542042	*Ctenopharyngodon idella*	grass carp	TLR2
KC816575	*Carassius carassius*	crucian carp	TLR2
AY388399	*Danio rerio*	zebrafish	TLR2a
NM_212812	*Danio rerio*	zebrafish	TLR2b
HQ677714	*Ictalurus punctatus*	channel catfish	TLR2
MG674831	*Plecoglossus altivelis*	ayu	TLR2a
GFIR01037304	*Plecoglossus altivelis*	ayu	TLR2b
HE979560	*Oncorhynchus mykiss*	rainbow trout	TLR2a
XM_021593972	*Oncorhynchus mykiss*	rainbow trout	TLR2b
XM_014200815	*Salmo salar*	Atlantic salmon	TLR2a
XM_014162243	*Salmo salar*	Atlantic salmon	TLR2b
XM_023989103	*Salvelinus alpinus*	Arctic char	TLR2b
KQ041805	*Larimichthys crocea*	large yellow croaker	TLR2 2a
KQ042333	*Larimichthys crocea*	large yellow croaker	TLR2 2b
FJ858800	*Cyprinus carpio*	commom carp	TLR2 2a
XM_019089843	*Cyprinus carpio*	commom carp	TLR2 2b
DQ012268	*Homo sapiens*	human	TLR2
AF216289	*Mus musculus*	house mouse	TLR2
CR533562	*Homo sapiens*	human	TLR1
AY009154	*Mus musculus*	house mouse	TLR1
GQ502184	*Oncorhynchus mykiss*	rainbow trout	TLR1
BC163271	*Danio rerio*	zebrafish	TLR1
FJ542041	*Ctenopharyngodon idella*	grass carp	TLR1
HQ677713	*Ictalurus punctatus*	channel catfish	TLR1
BC117422	*Homo sapiens*	human	TLR4
BC029856	*Mus musculus*	house mouse	TLR4
EU551724	*Danio rerio*	zebrafish	TLR4a
AY388400	*Danio rerio*	zebrafish	TLR4b
FJ542043	*Ctenopharyngodon idella*	grass carp	TLR4a
JF965431	*Ctenopharyngodon idella*	grass carp	TLR4b
HQ677716	*Ictalurus punctatus*	channel catfish	TLR4a
HQ677716	*Ictalurus punctatus*	channel catfish	TLR4b
DQ360816	*Homo sapiens*	human	TLR3
BC099937	*Mus musculus*	house mouse	TLR3
DQ459470	*Oncorhynchus mykiss*	rainbow trout	TLR3
AY616582	*Danio rerio*	zebrafish	TLR3
DQ864497	*Ctenopharyngodon idella*	grass carp	TLR3
HQ677715	*Ictalurus punctatus*	channel catfish	TLR3

**Table 3:** SRB sequences used in this study.

**Accession no. ensemble ID**	**Species**		**Protein**

	**Latin name**	**English name**
ENSTNIT00000001889	*mitochondrion Tetraodon nigroviridis*	spotted green pufferfish	SRB2b
XM_010737062	*Larimichthys crocea*	large yellow croaker	SRB2b
XM_021622581	*Oncorhynchus mykiss*	rainbow trout	SRB2b
JP736791	*Plecoglossus altivelis*	ayu	SRB2b
MH699854	*Ctenopharyngodon idella*	grass carp	SRB2b
XM_021468940	*Danio rerio*	zebrafish	SRB2b
NM_007644	*Mus musculus*	mouse	SRB2b
NM_005506	*Homo sapiens*	human	SRB2b
MH699855	*Plecoglossus altivelis*	ayu	SRB2a
XM_014160602	*Salmo salar*	Atlantic salmon	SRB2a
ENSTNIT00000018885	*mitochondrion Tetraodon nigroviridis*	spotted green pufferfish	SRB2a
NM_173259	*Danio rerio*	zebrafish	SRB2a
XM_019090575	*Cyprinus carpio*	common carp	SRB2a
MH699856	*Ctenopharyngodon idella*	grass carp	SRB2a
BC143319	*Homo sapiens*	human	SRB1
NM_016741	*Mus musculus*	mouse	SRB1
NM_198921	*Danio rerio*	zebrafish	SRB1
XM_011481418	*Oryziaslatipes*	Japanese rice fish	SRB1
XM_021590606	*Oncorhynchus mykiss*	rainbow trout	SRB1
NM_172604	*Mus musculus*	mouse	SRA3
NM_016240	*Homo sapiens*	human	SRA3
XM_004083965	*Oryziaslatipes*	Japanese rice fish	SRA3
LC107823	*Cyprinus carpio*	common carp	SRA3
XM_005160848	*Danio rerio*	zebrafish	SRA3

### Primary culture of ayu macrophages

After ayu were sacrificed, the head kidney was rapidly removed to isolate the macrophages. Ayu head kidney was washed in RPMI 1,640 medium supplemented with 2% fetal bovine serum (FBS; Invitrogen, Shanghai, China), penicillin (100 U/ml), streptomycin (100 μg/ml), and heparin (20 U/ml). The buffy coat cells were separated by using Ficoll Hypaque PREMIUM (1.077 ± 0.001 g/ml; Invitrogen) in combination with centrifugation according to the manufacturer's instructions. The cells were then seeded in 35 mm dishes at a density of 2 × 10^7^/ml. Non-adherent cells were washed off and the attached cells were incubated in complete medium (RPMI 1640, 5% ayu serum, 5% FBS, 100 U/ml penicillin, and 100 μg/ml streptomycin) at 24°C with 5% CO_2_. According to Giemsa staining results, over 96% of adherent cells were macrophages.

To inhibit clathrin-mediated endocytosis, sucrose hypertonic treatment was performed by incubating cells at 37°C with a 0.45 M sucrose solution; potassium (K^+^) depletion was performed by washing the cells at 37°C with PBS twice, followed by 5 min with hypotonic PBS (1:3 v/v with water), and five quick washes followed by 10 min incubation in K^+^-depletion solution (150 mM NaCl, 1 mM MgCl_2_, 1 mM CaCl_2_, 2 mM HEPES pH7.4, 0.5% DMSO, 0.5% BSA) (34). To inhibiting clathrin-independent endocytosis, we employed 5-(*N*-ethyl-*N*-isopropyl) amiloride (EIPA, 25 μm) and IPA-3 (IPA, 20 μm) to treat the cells (35).

### Real time quantitative PCR (RT-qPCR)

Total RNA was extracted from ayu tissues or macrophages using RNAiso reagents (TaKaRa, Dalian, China) for RT-qPCR. Each RNA sample (5 μg) was incubated with 1 U DNase I (Thermo Fisher Scientific, Grand Island, NY, USA) for 30 min at 37°C to remove residual genomic DNA. The first-strand cDNA was then synthesized using reverse transcriptase M-MLV (TaKaRa). Gene-specific primers for PaTLR2a, PaSRB2a, PaSRB2b, PaNOD1, PaNOD2, CD115, TNF, IL-1β, and housekeeping gene 18S rRNA are listed in Table 1. Gene 18S rRNA was utilized as an internal reference for normalization. The RT-qPCR reaction was performed using SYBR premix Ex Taq (Perfect Real Time, TaKaRa). The reaction mixture was incubated for 300 s at 95°C, followed by 35 amplification cycles of 30 s at 95°C, 30 s at 60°C, and 30 s at 72°C, on an ABI StepOne Real-Time PCR System (Applied Biosystems, Foster City, CA, USA). Ct values of PaTLR2a, PaSRB2a, PaSRB2b, PaNOD1, PaNOD2, TNF, and IL-1β for all samples were normalized to 18S rRNA using the 2^−ΔΔ*Ct*^ method.

### Enzyme-linked immunosorbent assay (ELISA)

LPS was diluted to the appropriate concentrations in PBS to infect ayu macrophages for different times and culture supernatant was collected for ELISA. Microplates (Nunc, Roskilde, Denmark) were coated overnight using concentrated cell supernatant. Plates were precoated using Poly-L-Lysine (Sigma Aldrich) to increase protein binding. Blocking of unbound binding sites was performed using 5% teleost gelatin (Sigma Aldrich). The anti-IL-1β and anti-TNF antibodies come from our previous studies (31, 34). One hundred microliters of 5 μg/ml anti-IL-1β or anti-TNF antibody was added to each well, incubated for 1 h, and washed three times. One hundred microliter of a 1:2500 dilution in PBS of a horseradish peroxidase (HRP)-labeled secondary antibody (Santa Cruz Biotechnology, Inc., Santa Cruz, CA, USA) in PBS was added to each well for 1 h and washed three times. Finally, alkaline phosphatase yellow liquid substrate system for ELISA (Sigma Aldrich) was used and optical density read at 405 nm.

### RNA interference (RNAi)

The target gene siRNA and scrambled siRNA of PaTLR2a,PaSRB2a, PaSRB2b, PaNOD1, and PaNOD2 for RNAi were designed and synthesized by Invitrogen, respectively. The siRNA sequences of target genes are listed in Table 1. Transfection of cells with siRNA was performed using the Lipofectamine 2,000 transfection reagent (Invitrogen) according to the manufacturer's protocol. Briefly, 5 μl of Lipofectamine 2,000 in 250 μl of Opti-MEM (Invitrogen) was mixed with either 100 pmol siRNA or 100 pmol scrambled siRNA in 250 μl of Opti-MEM. The mixture was then incubated for 20 min at room temperature before adding to macrophages with a final siRNA concentration of 40 nm. After a 5.5-h incubation, media were changed to complete media, and cells were cultured for another 48, 72, or 96 h before collection for expression analysis of PaTLR2a, PaSRB2a, PaSRB2b, PaNOD1, and PaNOD2. RT-qPCR and western blot analyses confirmed the knockdown of PaTLR2a, PaSRB2a, PaSRB2b, PaNOD1, and PaNOD2 mRNA and protein, respectively.

### Western blot

Ayu macrophages were washed twice in PBS and lysed in a buffer [20 mM HEPES [pH7.4], 1.5 mM MgCl_2_, 0.2 mM EDTA, 100 mM NaCl, 0.2 mM DTT, 0.5 mM sodium orthovanadate, and 0.4 mM PMSF] containing phosphatase inhibitor (Phosphatase Inhibitor Cocktail, Sigma Aldrich). The protein concentration of the supernatant was measured in each soluble fraction using the Bradford method, and samples were subjected to SDS-PAGE (12% acrylamide gel) and transferred to PVDF (Pall, New York, NY, USA). Membranes were blocked for 1 h in a 10% non-fat dry milk solution in TBS-Tween. After a 1.5-h incubation with antibodies against PaTLR2a, PaSRB2a, PaSRB2b, PaNOD1, or PaNOD2, membranes were washed and incubated for 1 h with HRP-labeled secondary antibody (1:5000, Santa Cruz Biotechnology). Proteins were visualized by enhanced chemiluminescence (Santa Cruz Biotechnology). Ayu β-actin was used as the control. The intensity of each band obtained by western blot was analyzed using the NIH ImageJ program.

The antibodies of PaTLR2a, PaSRB2a, and PaSRB2b were prepared using peptides derived from these proteins (PaTLR2a: 123-139 aa, PaSRB2a: 243-259 aa, and PaSRB2b: 336-352 aa, GL Biochem, Shanghai, China). These peptides were synthesized to generate the respective monoclonal antibodies (GL Biochem; 1 mg/ml). The antibodies were validated using flow cytometry and Western blot (Supplemental Figures 1A–C). For flow cytometry, the antibodies to PaTLR2a, PaSRB2a, and PaSRB2b were used at dilutions of 1:200, 1:300, and 1:200, respectively. For western blotting, antibodies to PaTLR2a, PaSRB2a, and PaSRB2b were used at dilutions of 1:400, 1:600, and 1:400, respectively. Antibodies to PaNOD1 and PaNOD2 were prepared using recombinant proteins. The recombinant plasmids were constructed and transformed into *E. coli* BL21 (DE3) for over-expression. PaNOD1 and PaNOD2 were expressed as inclusion bodies and were purified using a Ni-NTA column (QIAGEN, Shanghai, China), according to the manufacturer's instructions. Endotoxin in the recombinant proteins was < 0.1 EU/mg after processing with an endotoxin-removal column (Pierce, Rockford, IL). The concentration of the PaNOD1 and PaNOD2 antibodies were both 2.5 mg/ml quantified by the Bradford method. The primer sequences for the target genes are listed in Table 1. The antibodies were prepared from the rabbit against PaNOD1 and PaNOD2. The specificity of anti-PaNOD1 and anti-PaNOD2 antibodies was validated using ELISA and Western blot (Supplemental Figures 1D,E). For ELISA, the antibodies to PaNOD1 and PaNOD2 were used at dilutions of 1:300 and 1:400, respectively. For Western blot, antibodies to PaNOD1 and PaNOD2 were used at dilutions of 1:600 and 1:800, respectively.

### LPS uptake experiments

Macrophages or HEK293T cells were incubated at 24°C with FITC-LPS (Sigma Aldrich). After incubation, cells were washed with PBS and further treated with 250 μg/ml proteinase K (Sigma Aldrich) for 30 min to remove cell surface-bound LPS. The remaining LPS were considered to be intracellular. FITC-LPS in macrophages was measured by a Gallios flow cytometer (Beckman Coulter, Miami, FL, USA). To evaluate the participation of scavenger receptors in LPS uptake, cells were pretreated with oxLDL and polyG for 30 min before measurement of FITC-LPS uptake.

For confocal microscopy analysis, cells were washed three times with PBS and fixed with 200 μl of 4% paraformaldehyde in PBS for 30 min after LPS internalization, then air-dried. After three washes with PBS, cells were probed with rhodamine phalloidin for 30 min (66 nm, Invitrogen) to indicate filamentous cell membrane (red). DAPI (10 μg/ml, Sigma-Aldrich, Shanghai, China) was used to stain the cell nucleus for 5 min after washing with PBS. Coverslips were viewed using an IX81-FV1000 microscope (Olympus, Tokyo, Japan).

### DNA constructs

Chimeric DNA constructs of PaSRB2a and PaSRB2b were created using a standard overlap extension PCR technique. Chimeric PCR products were cloned into the pcDNA3.1 vector. Sequencing was performed for all DNA constructs, which were used to transfect HEK293T cells. For PaSRB2a overexpression in macrophages, PaSRB2a was prepared using a PCR-based method. For expression, a lentiviral vector carrying cDNA of PaSRB2a was constructed using the ViraPower lentiviral expression system (Invitrogen). Viral particles were produced in HEK293T cells according to the manufacturer's instructions. Cells were treated with blasticidin for selecting cells with successful integration of the plasmid. Lentiviral vectors were transduced into ayu macrophages at a multiplicity of infection of three.

### Reporter gene assays

HEK293T cells were co-transfected with PaSRB2a, PaNOD1, or PaNOD2 luciferase reporter vector, NF-κB luciferase reporter plasmid (or IFNβ luciferase reporter plasmid), and *Renilla* luciferase plasmid. After 24 or 48 h, the cells were collected and assayed for reporter activity using the Dual-Luciferase reporter system following the manufacturer's instructions (Promega, Shanghai, China). The relative luciferase activity was achieved against the *Renilla* luciferase control.

### LPS precipitation assay

The polysaccharide moiety of LPS was biotin-labeled using biotin hydrazide (Thermo Fisher Scientific) according to the manufacturer's instructions. HEK293T cells were transfected with PaSRB2a, PaNOD1, or PaNOD2. Cells were washed and lysed for immunoprecipitations. The cell lysates were incubated with biotinylated LPS at 4°C overnight. Biotinylated LPS protein complexes were collected by addition of 20 μl of packed streptavidin beads. The presence of PaSRB2a, PaNOD1, or PaNOD2 in the LPS complexes was assessed by western blotting.

### Knockdown with lentivirus *in vivo*

For gene knockdown *in vivo* by lentivirus delivery, the small interfering RNAs (siRNAs) against PaSRB2a were predicted by BLOCK-iT RNAi Designer (Invitrogen). Short hairpin RNAs containing the selected siRNA sequences, as well as scrambled siRNA, were designed and listed in Table 1. DNA oligonucleotides for short hairpin RNA expression were synthesized by Invitrogen, annealed, and constructed into pSUPER vector (Oligoengine, Seattle, WA, USA) downstream of the H1 promoter, as previously described (36). The generated constructs with different siRNAs (1 μg) or control pSUPER (1 μg) along with overexpression plasmid pcDNA3.1-PaSRB2a (1 μg) were co-transfected into HEK293T cells in 12-well plates. The efficiency of siRNAs against PaSRB2a was determined by RT-qPCR.

The U6 promoter cassette in lentiviral vector pLB was replaced by the H1-siRNA cassette excised from the most effective siRNA constructs to produce lentiviral vectors. Lentiviruses were produced by transient transfection of packaging cell line HEK293T cells. In brief, Lentiviral vectors (20 μg) were co-transfected with pCMV-dR8.2 dvpr (15 μg) and pCMV-VSV-G (6 μg) packaging vectors into HEK293T cells using FuGENE 6 Transfection Reagent (Promega). The lentiviral supernatant was harvested at 48–72 h post transfection, concentrated via ultracentrifugation at 25,000 rpm for 90 min at 4°C (SW-41Ti rotor) to dissolve in 100 μl PBS, and then purified and concentrated using the Fast-Trap Lentivirus Purification and Concentration Kit (Millipore, Bedford, MA, USA). Lentivirus titers were determined by transduction analysis of GFP expression in HEK293T cells. Infected cells were examined under a fluorescent microscope investigation (Nikon, Tokyo, Japan).

The silencing efficiency of the constructed lentivirus was determined both *in vitro* and *in vivo*. *In vitro*, HEK293T cells were transfected with pcDNA3.1-PaSRB2a. Concentrated lentivirus (10 μl) was added to the culture medium and 4 μg/ml of polybrene (Sigma Aldrich) was simultaneously added to increase the infection efficiency. The silencing efficiency of lentivirus was detected by RT-qPCR. For *in vivo* assay, the lentiviruses (1 × 10^8^ TU/fish/day) was repeatedly delivered into fish by injection once a day for 3 d. Total RNA from liver and spleen were isolated and reverse transcribed into cDNA. RT-qPCR was conducted to evaluate the efficiency of *in vivo* suppression of PaSRB2a.

### Survival assay

Survival rate assay was performed as previously described (37). Briefly, fish were divided into different treatment groups, each containing 30 fish. The fish in the experimental groups received ip. injections of LPS with 0.2 mg/g body weight, while the control group received PBS. Morbidity was monitored for 7 days after challenge, and the results were recorded every 24 h.

### *In vivo* macrophage depletion

Clondronate-liposome suspension has been given to deplete tissue macrophages in rodents and some kinds of teleosts (37, 38). Ayu macrophage depletion was performed by ip injection with clodronate-liposomes or PBS-liposomes on days −4 and −2. The clodronate-liposomes were given ip at a dose of 0.25 ml/100 g. CD115 has been identified as a marker of monocytes/macrophages in mammal and teleosts (39). The depletion of monocytes/macrophages was confirmed by flow cytometry analysis of CD115-positive cells in the blood and RT-qPCR analysis of CD115 mRNA in peripheral blood mononuclear cells 4 days after first injection of liposomes (37). Peripheral blood mononuclear cells were isolated from freshly collected blood by Ficoll (Invitrogen) density gradient centrifugation, washed, and resuspended in PBS. Antibodies to ayu CD115 were prepared at 1 mg/ml in previous work (37) and used at a dilution of 1:300 for flow cytometry.

### Statistical analysis

Data are presented as mean ± SEM. The animals used in the experiments were randomly chosen. Animal experiments were performed by an observer blinded to the experimental conditions. The survival curves were analyzed using the Kaplan-Meier method. All other data were analyzed by one-way ANOVA. When variances were significantly different (*p* < 0.05), logarithmic transformation was used to stabilize the variance. ^*^, ^**^, and ^***^ represent *p*-values, 0.05, 0.01, and 0.001, respectively.

## Results

### LPS affects cytokine production in macrophages

It has been reported that high concentrations of LPS are required to activate leukocytes from several types of teleosts *in vitro* (7, 40). We measured TNF and IL-1β mRNA expression in ayu macrophages at different time points after LPS treatment at 10 μg/ml. TNF and IL-1β were both up-regulated after LPS O55:B5 treatment (Supplemental Figures 2A,B). We further measured TNF and IL-1β mRNA expression in macrophages at different LPS concentrations. LPS treatment induced a dramatic up-regulation of TNF and IL-1β mRNA expression at 10 and 100 μg/ml (Supplemental Figures 2C,D). There was no up-regulation of TNF and IL-1β observed in LPS-treated macrophages with concentrations ≤ 1 μg/ml. Up-regulation of TNF and IL-1β proteins were found at 4, 8, and 16 h after LPS treatment at 10 μg/ml (Supplemental Figures 2E,F). These data suggest that LPS initiates inflammatory responses in teleost macrophages at high concentrations.

### TLR2 does not mediate the LPS effect in ayu macrophages

TLR4 activation by LPS does not lead to a pro-inflammatory reaction in zebrafish (7). However, it is still unclear whether TLR2 mediates LPS signaling in teleost macrophages. We first cloned the PaTLR2a and PaTLR2b genes from ayu. PaTLR2a and PaTLR2b associated with the TLR2 genes from other animals to form a cluster in a phylogenetic tree (Supplemental Figure 3). PaTLR2a was expressed in ayu macrophages (Supplemental Figures 4A,B). However, PaTLR2b could not been detect in ayu macrophages by RT-qPCR. Moreover, PaTLR2b is non-existent in the transcriptome of ayu macrophages (35). We further investigate whether PaTLR2a mediates LPS effect in ayu macrophages. We knocked down PaTLR2a gene in ayu macrophages by RNAi (Supplemental Figures 4A,B). The PaTLR2a mRNA was down-regulated at 48, 72, and 96 h after PaTLR2a siRNA treatment in ayu macrophages (Supplemental Figure 4A). Western blot results confirmed that PaTLR2a protein was down-regulated at 96 h after PaTLR2a siRNA treatment in macrophages (Supplemental Figure 4B). We further investigated the effect of PaTLR2a on TNF and IL-1β expression. PaTLR2a siRNA treatment did not affect TNF and IL-1β expression in macrophages treated with LPS (Supplemental Figures 4C,D).

### LPS enters ayu macrophages by internalization

**Figure 1:**
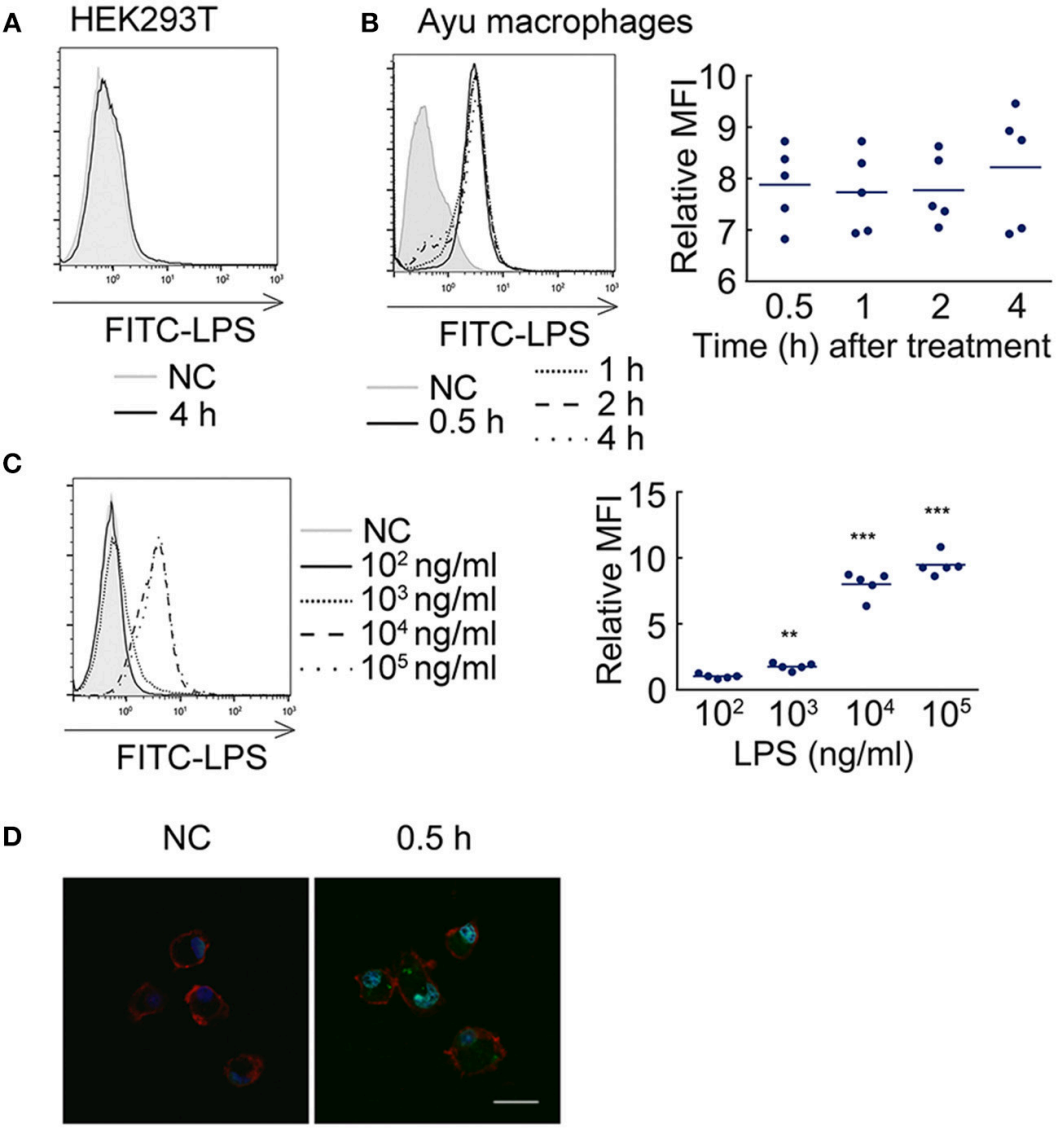
LPS internalization in ayu macrophages. **(A)** LPS did not enter HEK293T cells after 4 h incubation with 10 μg/ml LPS. **(B)** LPS detection in ayu macrophages after LPS treatment at different time points. The LPS concentration was 10 μg/ml. **(C)** LPS detection in ayu macrophages after LPS treatment for 4 h at different LPS concentrations. **(D)** Internalization of LPS in ayu macrophages was observed by confocal microscopy. Green color: FITC-LPS, red color: rhodamine-phalloidin, blue color: 4', 6'-diamidino-2-phenylindole (DAPI). Bar, 20 μm. Each bar represents the mean ± SE. *n* = 5. Data are representative of two **(A–C)** and three **(D)** independent experiments. **P < 0.01, ***P < 0.001.

Since TLR2 also does not mediate the effect of LPS on the pro-inflammatory reaction, we further asked whether LPS can enter ayu macrophages to induce pro-inflammatory responses. First, we measured whether LPS can enter HEK293T cells. FITC-LPS were incubated with HEK293T cells for 4 h, and proteinase K was used to remove extracellular LPS. No FITC-LPS signaling was detected by flow cytometry in HEK293T cells (Figure 1A). We further collected ayu macrophages at different time points after FITC-LPS treatment. Specific internalization of LPS was detected by flow cytometry (Figure 1B). Since high concentrations of LPS are required to activate teleost leukocytes, we further measured LPS internalization in macrophages with different LPS concentrations. The MFI of internalization of LPS at 1 μg/ml was 1.7-fold that at 10^3^ ng/ml, while the MFI of internalization of LPS at 10 μg/ml was 7.8-fold that at 10^4^ ng/ml (Figure 1C). Furthermore, confocal microscopy indicated that LPS was internalized within the cell cytoplasm 30 min after incubation (Figure 1D).

**Figure 2:**
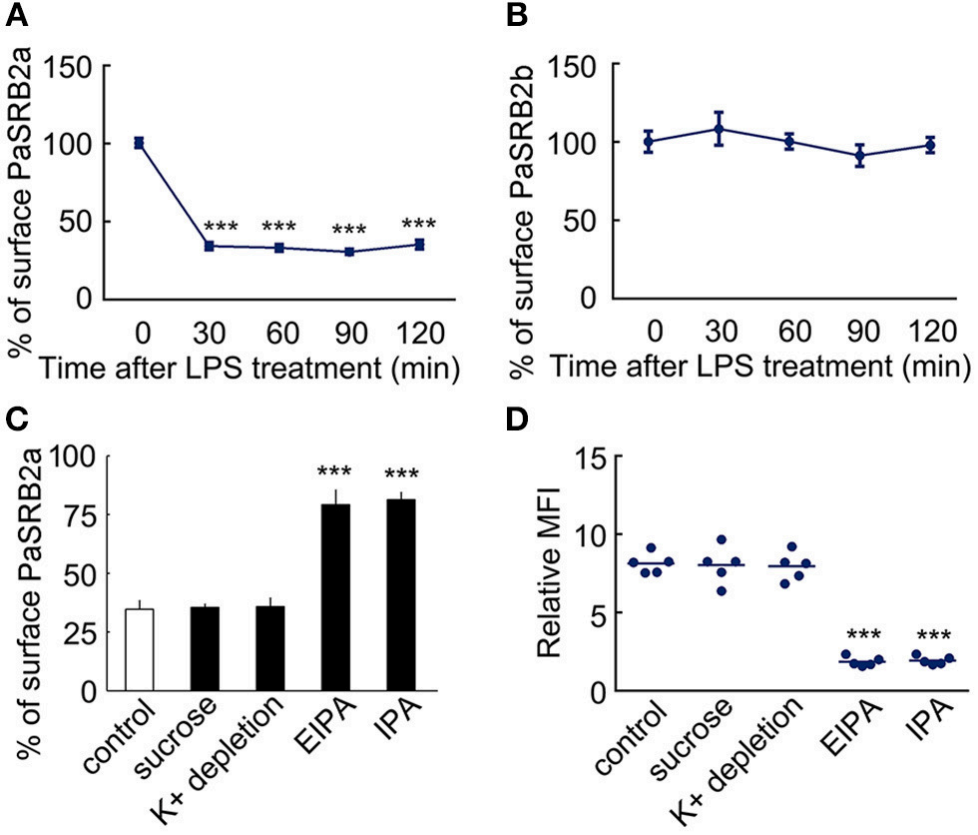
LPS internalization accompanied PaSRB2a endocytosis in ayu macrophages. **(A)** Surface levels of PaSRB2a were measured by flow cytometry at different time points after treatment with 10 μg/ml LPS. **(B)** Surface levels of PaSRB2b were measured by flow cytometry at different time points. **(C)** Surface levels of PaSRB2a after treatment with chemical clathrin-mediated endocytosis inhibitors (sucrose and K^+^ depletion) or clathrin-independent endocytosis inhibitors (EIPA, 5-(*N*-ethyl-*N*-isopropyl) amiloride and IPA, IPA-3). **(D)** LPS internalization after treatment with membrane endocytosis inhibitors. Each bar represents the mean ± SE. *n* = 5. Data are representative of three independent experiments. ***P < 0.001.

It is well-known that scavenger receptors mediate the recognition and internalization of endotoxin, not only in mammals (41), but also in teleosts (42). We further investigated what types of scavenger receptors mediated LPS internalization. First, we employed antagonists of scavenger receptor B (SRB, oxLDL) and scavenger receptor A (SRA, polyG) to measure their effect on LPS internalization. OxLDL reduced the MFI of FITC-LPS in ayu macrophages (MFI: 8.1 at 0 μg/ml, 3.2 at 10 μg/ml, 1.8 at 50 μg/ml, respectively). PolyG did not affect LPS internalization in macrophages at 1 and 5 mg/ml. These data suggest that SRB may participate in LPS internalization in macrophages. We then cloned two SRB genes from ayu macrophages, defined as PaSRB2a and PaSRB2b. The receptor internalization of PaSRB2a and PaSRB2b was measured after LPS treatment by flow cytometry and western blot. LPS treatment at different time points led to the internalization of PaSRB2a, but not PaSRB2b (Figures 2A,B). PaSRB2a and LPS internalization was not affected after sucrose hypertonic treatment and K^+^ depletion, which both inhibit clathrin-mediated endocytosis (Figures 2C,D). However, PaSRB2a and LPS internalization was prevented after treatment with 5-(*N*-ethyl-*N*-isopropyl) amiloride (EIPA) or IPA-3 (IPA), which inhibit clathrin-independent endocytosis (Figures 2C,D). These data suggest that PaSRB2a may play an important role in LPS internalization by ayu macrophages.

### PaSRB2a mediates LPS internalization

**Figure 3:**
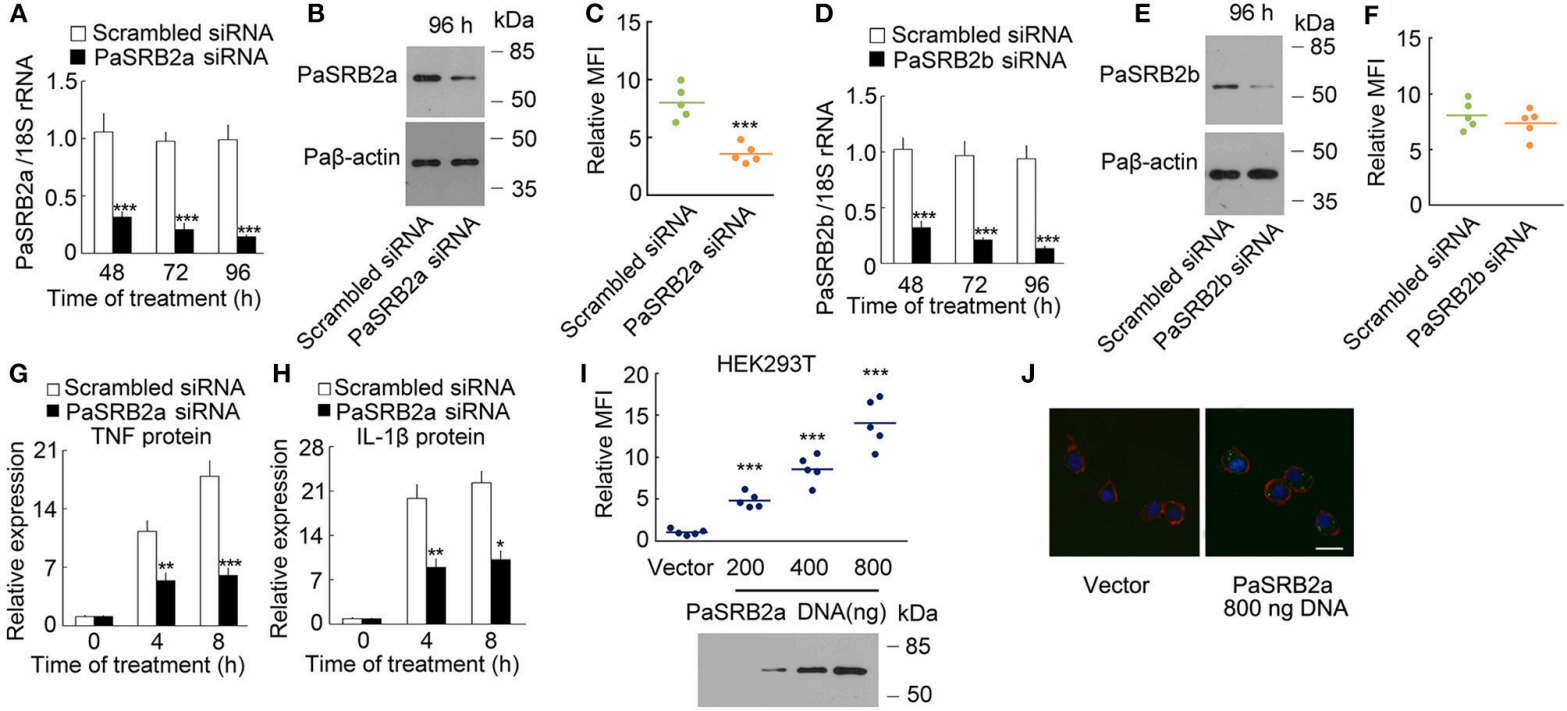
PaSRB2a mediated LPS internalization and pro-inflammatory response. **(A)** PaSRB2a mRNA levels in ayu macrophages treated with PaSRB2a siRNA. **(B)** PaSRB2a protein levels in ayu macrophages treated with PaSRB2a siRNA. Representative blots of three independent experiments are shown. **(C)** Effect of PaSRB2a siRNA on LPS internalization in ayu macrophages. **(D)** PaSRB2b mRNA levels in ayu macrophages treated with PaSRB2b siRNA. **(E)** PaSRB2b protein levels in ayu macrophages treated with PaSRB2b siRNA. Representative blots of three independent experiments are shown. **(F)** Effect of PaSRB2b siRNA on LPS internalization in ayu macrophages. **(G)** PaSRB2a effect on TNF expression in LPS-treated ayu macrophages. **(H)** PaSRB2a effect on IL-1β expression in LPS-treated ayu macrophages. **(I)** LPS internalization in HEK293T cells transfected with a PaSRB2a expression plasmid by flow cytometry. Representative blots of three independent experiments are shown. (**J**) Internalization of LPS in HEK293T was observed by confocal microscopy. Green color: FITC-LPS, red color: rhodamine-phalloidin, blue color: 4', 6'-diamidino-2-phenylindole (DAPI). Bar, 20 μm. Each bar represents the mean ± SE. *n* = 5. Data are representative of two **(A,C,D,F–I)** and three **(B,E,J)** independent experiments. *P < 0.05, **P < 0.01, ***P < 0.001.

To confirm the effect of PaSRB2a on LPS internalization, we knocked down PaSRB2a in ayu macrophages by siRNA. The PaSRB2a mRNA was down-regulated at all tested time points after PaSRB2a siRNA treatment in ayu macrophages (Figure 3A). Western blot results confirmed that PaSRB2a protein was down-regulated at 96 h after PaSRB2a siRNA treatment in macrophages (Figure 3B). PaSRB2a siRNA treatment reduced LPS internalization in macrophages (Figure 3C). Furthermore, PaSRB2b siRNA treatment also knocked down the expression of PaSRB2b in macrophages (Figure 3D). Western blot results confirmed that PaSRB2b protein was down-regulated at 96 h after PaSRB2b siRNA treatment in macrophages (Figure 3E). However, there was no change in LPS internalization observed in macrophages after PaSRB2b siRNA treatment (Figure 3F). We further measured whether the internalization of LPS mediated by PaSRB2a affects TNF and IL-1β production. TNF and IL-1β protein levels at both 4 and 8 h were lower in macrophages treated with PaSRB2a siRNA than in those treated with scrambled siRNA (Figures 3G,H). Moreover, PaSRB2a was transfected into HEK293T cells with no LPS internalization before transfection. LPS internalization was then observed in HEK293T cells after transfection with PaSRB2a (Figures 3I, J). Our results demonstrate that PaSRB2a mediates TNF and IL-1β production after LPS induction in ayu macrophages by regulating LPS internalization.

### PaNOd1 and paNOD2 also mediate the LPS effect

**Figure 4:**
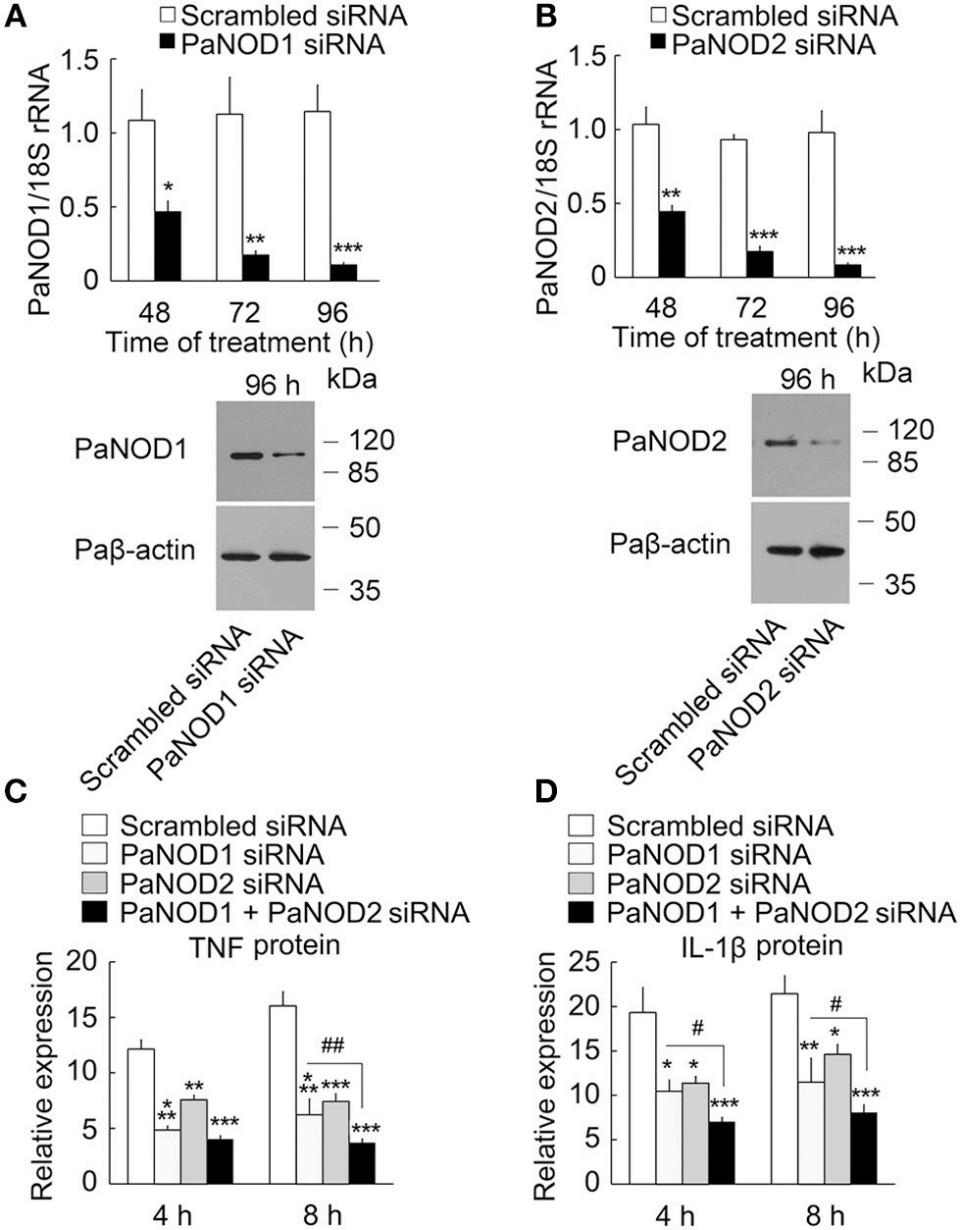
Effect of PaNOD1 and PaNOD2 on the production of TNF and IL-1β in ayu macrophages. **(A)** PaNOD1 mRNA and protein expression in macrophages treated with PaNOD1 siRNA. Representative blots of three independent experiments are shown. **(B)** PaNOD2 mRNA and protein expression in macrophages treated with PaNOD2 siRNA. Representative blots of three independent experiments are shown. **(C)** TNF production in macrophages after PaNOD1 siRNA, PaNOD2 siRNA, or PaNOD1 plus PaNOD2 siRNA treatment. **(D)** IL-1β expression in macrophages after PaNOD1 siRNA, PaNOD2 siRNA, or PaNOD1 plus PaNOD2 siRNA treatment. Each bar represents the mean ± SE. *n* = 5. Data are representative of three independent experiments. *P < 0.05, **P < 0.01, ***P < 0.001. ^#^*P* < 0.05, ^*##*^*P* < 0.01 vs. PaNOD1 siRNA.

We then determined the presence of an intracellular receptor for LPS. NOD-like receptors play an important role in host defense as intracellular receptors for pathogen-associated molecular patterns (28, 43). We cloned the nucleotide-binding oligomerization domain 1 (PaNOD1) and PaNOD2 genes from ayu macrophages, and employed RNAi to knockdown the expression of NOD1 and NOD2 in ayu macrophages. Both PaNOD1 and PaNOD2 siRNAs reduced the RNA levels of PaNOD1 and PaNOD2 in ayu macrophages at 48, 72, and 96 h after siRNA treatment (Figures 4A,B). Western blot results confirmed that PaNOD1 and PaNOD2 protein levels were down-regulated at 96 h after PaNOD1 and PaNOD2 siRNA treatment in macrophages (Figures 4A,B). TNF and IL-1β were measured in LPS-treated macrophages after siRNA incubation. Both PaNOD1 and PaNOD2 siRNA incubation resulted in the down-regulation of TNF and IL-1β protein expression in macrophages at 4 and 8 h after LPS treatment (Figures 4C,D).

### Critical domains for paSRB2a-dependent LPS internalization

**Figure 5:**
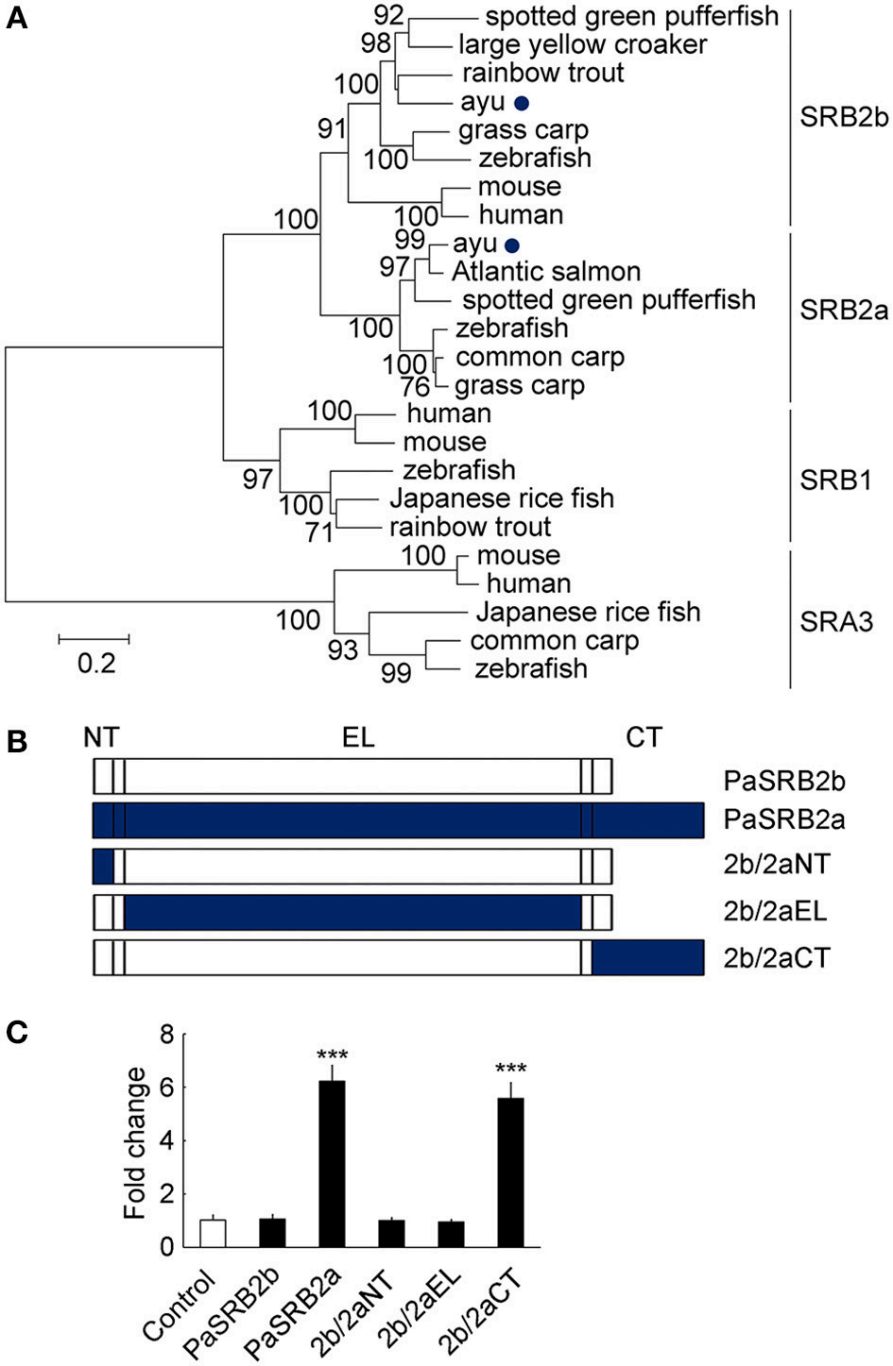
The critical domains for PaSRB2a uptake of LPS. **(A)** Phylogenetic (neighbor-joining) analysis of SRB amino acid sequences using MEGA 5.0. Node values represent the percentage bootstrap confidence derived from 1,000 replicates. The accession numbers of scavenger receptor genes are listed in Table 3. **(B)** Organization of cDNAs encoding PaSRB2a and PaSRB2b chimeras. PaSRB2a and PaSRB2b are predicted to have an intracellular N terminus, large extracellular domain, and intracellular C terminus. **(C)** LPS internalization in HEK293T cells transfected with PaSRB2a-PaSRB2b chimeras. 2b/2aNT: PaSRB2b-PaSRB2a N-terminal domain chimera, 2b/2aEL: PaSRB2b-PaSRB2a extracellular loop domain chimera, 2b/2aCT: PaSRB2b-PaSRB2a C-terminal chimera. Data are expressed as fold-change compared to the control. ***P < 0.001.

We found that ayu PaSRB2a and PaSRB2b were clustered together in a phylogenetic tree built with the SRBS genes from several other animals (Figure 5A). PaSRB2a and PaSRB2b share a high degree of sequence similarity. However, PaSRB2a, but not PaSRB2b, mediated LPS internalization in ayu macrophages. Thus, we expressed a chimeric PaSRB2a and PaSRB2b receptor to determine the domains of PaSRB2a responsible for LPS internalization (Figure 5B). When we expressed the chimeric receptor consisting of the N-terminal intracellular and extracellular domains of PaSRB2b and the C-terminal intracellular domain of PaSRB2a, we detected a increase in LPS internalization (Figure 5C), suggesting that the C-terminal intracellular domain of PaSRB2a plays an important role in LPS internalization.

### The pathway NF-κB activation by LPS

**Figure 6:**
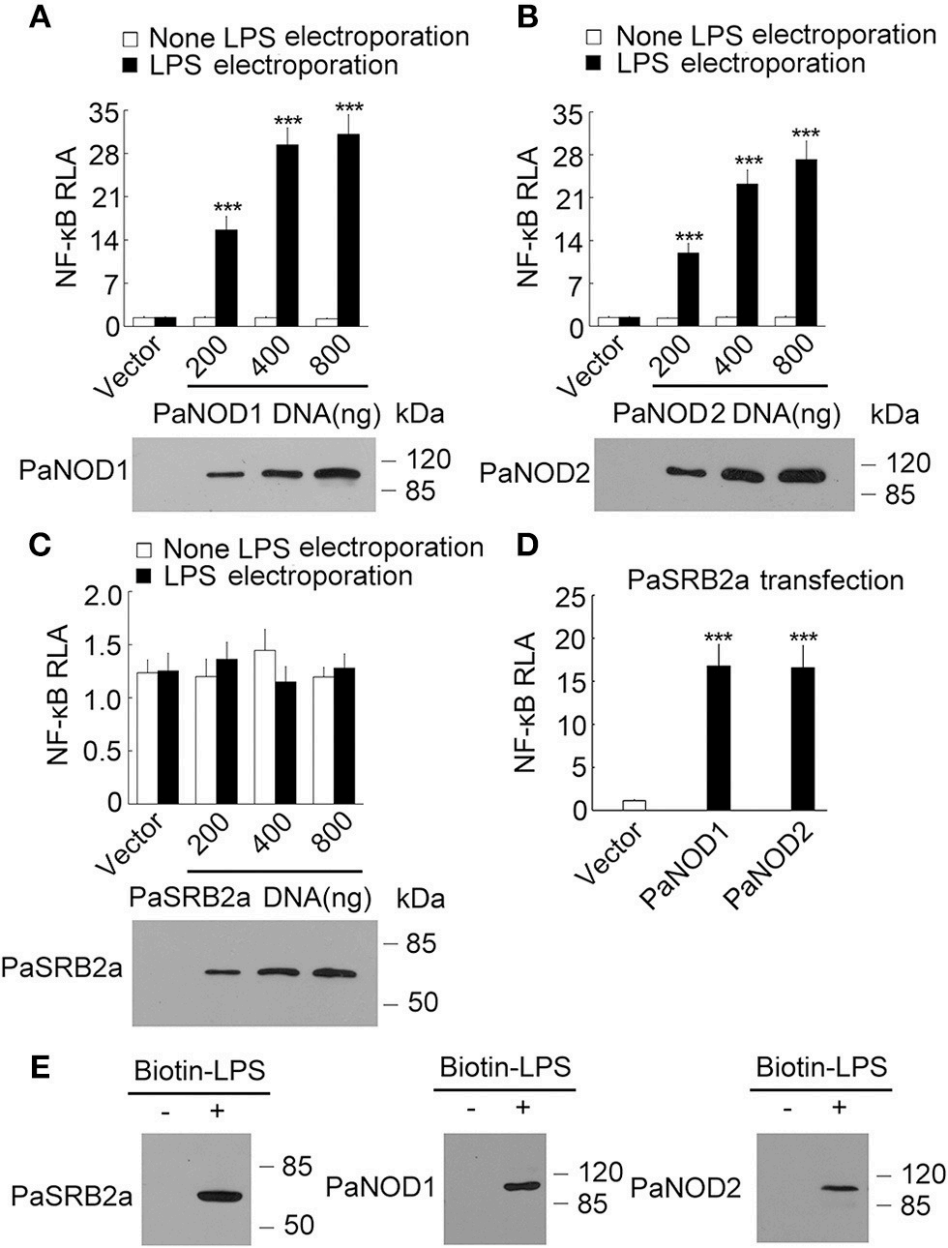
LPS activated NF-κB via PaNOD1, PaNOD2, and PaSRB2a. **(A)** PaNOD1 effect on NF-κB activation in LPS-treated HEK293T cells. **(B)** PaNOD2 effect on NF-κB activation in LPS-treated HEK293T cells. **(C)** PaSRB2a effect on NF-κB activation in LPS-treated HEK293T cells. **(D)** The effect of PaNOD1 and PaNOD2 in HEK293T cells with PaSRB2a transfection after LPS treatment. Bottom panels present western blots of cell lysates to monitor protein expression. **(E)** Physical interaction between LPS and PaSRB2a, PaNOD1, PaNOD2. Each lane represents the cell lysate incubated with biotinylated LPS (Biotin-LPS) and avidin beads. Each bar represents the mean ± SE. Representative blots of three independent experiments are shown. *n* = 5. Data are representative of two **(A–D)** and three **(E)** independent experiments. ***P < 0.001.

Our results illustrate that PaSRB2a, PaNOD1, and PaNOD2 were important in the LPS-mediated pro-inflammatory reaction in ayu macrophages. We then attempted to determine whether PaNOD1 and PaNOD2 act downstream of PaSRB2a. HEK293T cells were transfected with increasing amounts of PaNOD1 to measure the effect of LPS on NF-κB activation. The relative luciferase activity of NF-κB was not affected in PaNOD1-transfected HEK293T cells after LPS incubation without electroporation (Figure 6A). The relative luciferase activity was dramatically up-regulated after LPS electroporation (Figure 6A). PaNOD2 transfection also led to the up-regulation of relative luciferase activity of NF-κB in HEK293T cells with LPS electroporation, but not in HEK293T cells without LPS electroporation (Figure 6B). The relative luciferase activity of NF-κB was not affected by PaSRB2a transfection in LPS-treated HEK293T cells with or without electroporation (Figure 6C). Furthermore, both PaNOD1 and PaNOD2 increased the relative luciferase activity of NF-κB after LPS incubation without electroporation in PaSRB2a transfected HEK293T cells (Figure 6D). The relative luciferase activity of IFNβ was not changed after LPS incubation in PaSRB2a-, PaNOD1-, and PaNOD2-transfected HEK293T cells (Supplemental Figure 5). We further investigated if PaSRB2a, PaNOD1, and PaNOD2 directly bind LPS. LPS precipitation assay illustrated that LPS interacted physically with PaSRB2a, PaNOD1, and PaNOD2 (Figure 6E). These data suggest that PaSRB2a is necessary for LPS internalization to activate inflammatory responses.

### PaSRB2a mediate the effect of LPS *in vivo*

**Figure 7:**
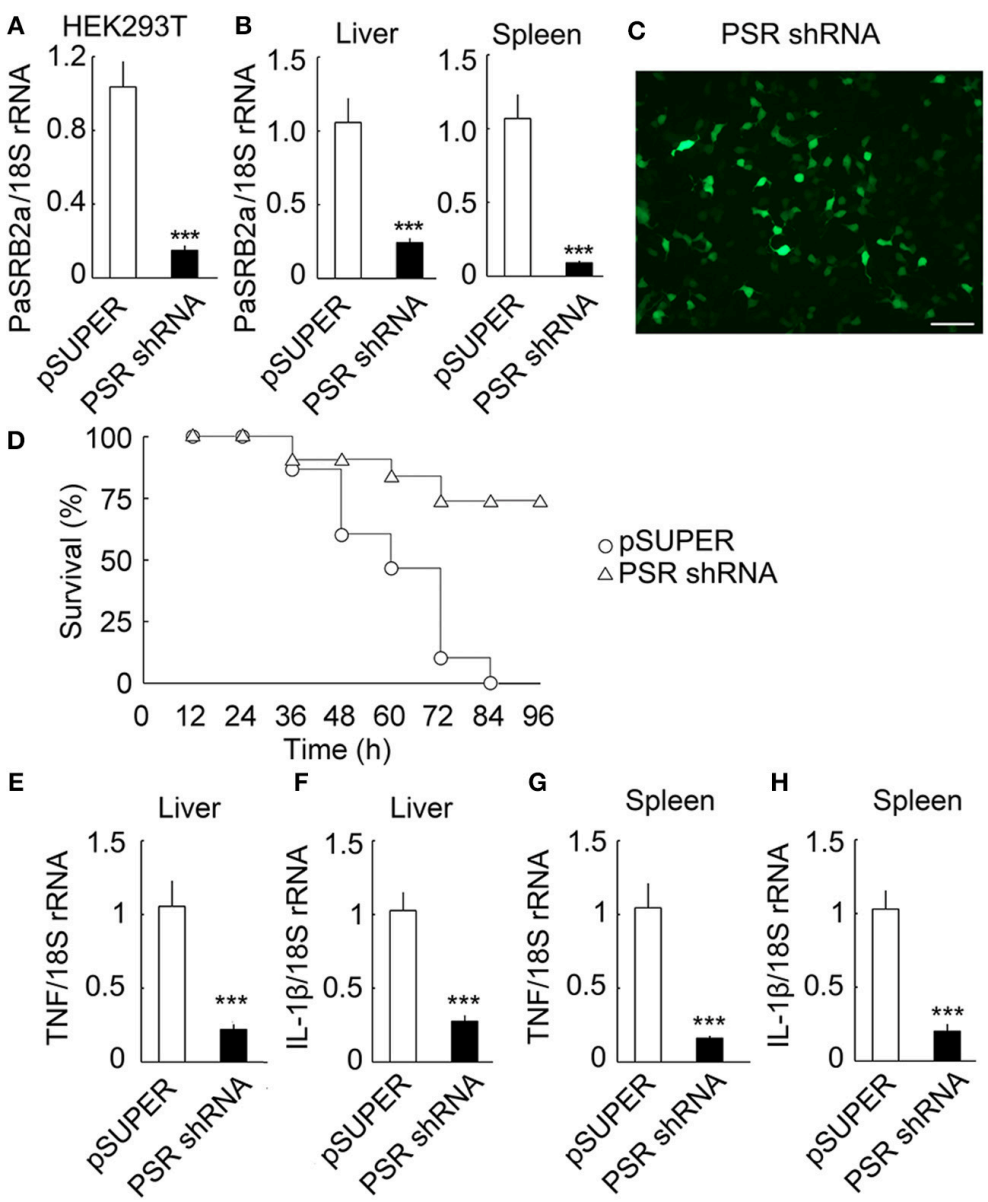
Effect of PaSRB2a on outcome of LPS-treated ayu. **(A)** PaSRB2a shRNA decreased the expression of PaSRB2a in HEK293T cells. **(B)** PaSRB2a mRNA expression in liver and spleen after delivery of lentiviruses into ayu. **(C)** shRNA expression efficiency of lentiviruses. Bar: 100 μm. GFP fluorescence was detected in HEK293T cells by microscopy. **(D)** Survival in ayu treated with pSUPER and pSUPER-PaSRB2a shRNA. *n* = 30 per group. RT-qPCR analysis of TNF **(E)** and IL-1β **(F)** expression in ayu liver. RT-qPCR analysis of TNF **(G)** and IL-1β **(H)** expression in ayu spleen. PSR shRNA: PaSRB2a shRNA. Each bar represents the mean ± SE. *n* = 5. Data are representative of two **(A,B, E–H)** and three **(C,D)** independent experiments. ***P < 0.001.

We further investigated PaSRB2a function *in vivo* by delivering lentivirus into ayu tissues. Using HEK293T cells, we identified the most effective siRNA (85.5%) for pSUPER-PaSRB2a (Figure 7A). The interference efficiency of this system for PaSRB2a reached 77.1% in liver and 91.5% in spleen (Figure 7B). The siRNA expression efficiency of the lentiviruses detected in HEK293T cells reached > 90% using the expression of GFP as an indicator for PaSRB2a (Figure 7C). The survival rates of pSUPER-PaSRB2a-treated ayu were up-regulated at 96 h after LPS administration compared with pSUPER empty vector (Figure 7D). In pSUPER-PaSRB2a-transfected ayu, the TNF and IL-1β mRNA levels in liver were down-regulated compared with those in pSUPER empty vector transfected ayu (Figures 7E, F). Both TNF and IL-1β were also down-regulated in the spleen in pSUPER-PaSRB2a-transfected ayu compared with those in pSUPER-transfected ayu (Figures 7G,H).

### PaSRB2a mediates the effect of LPS pre-stimulation on pro-inflammatory responses

**Figure 8:**
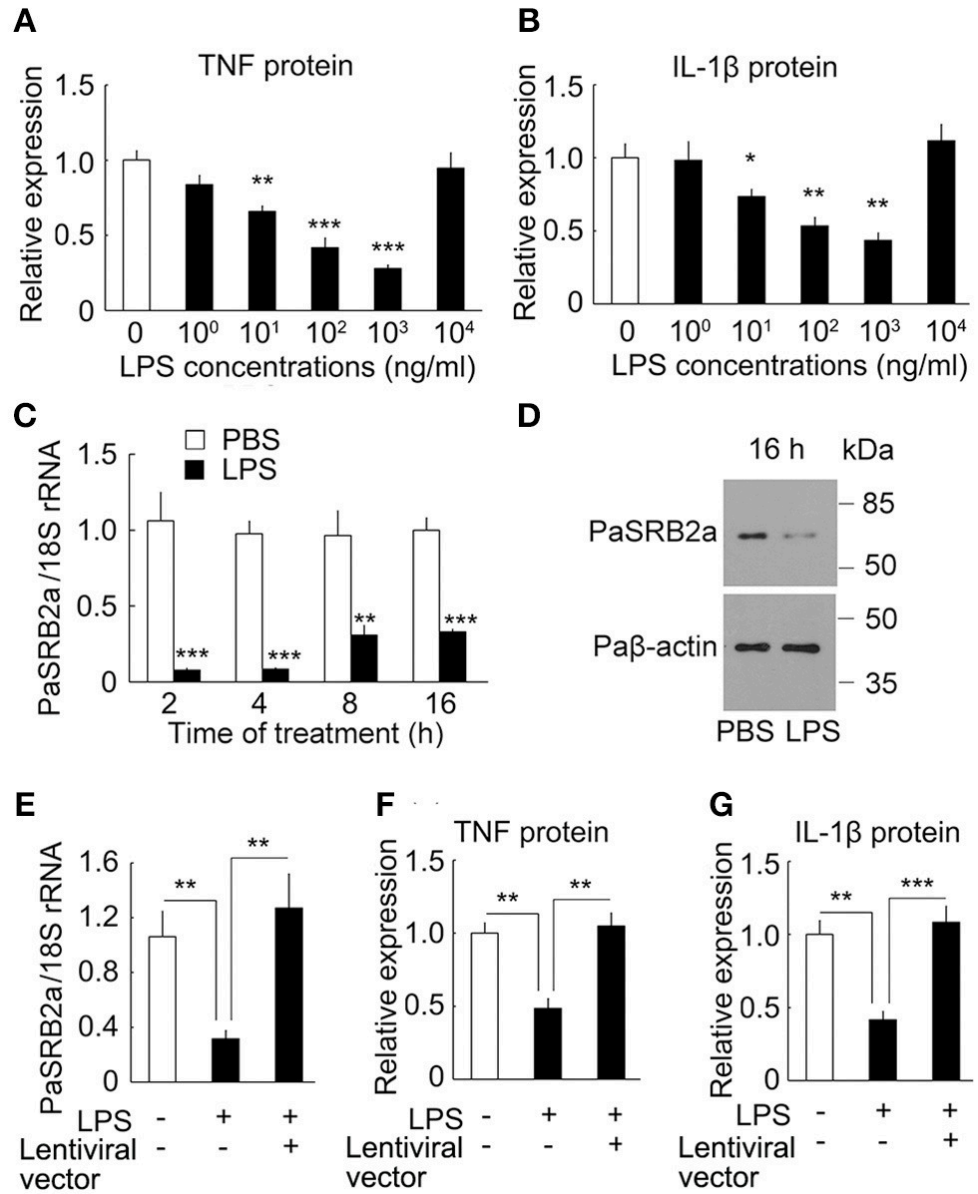
PaSRB2a mediated the effect LPS pre-stimulation on the expression of pro-inflammatory cytokines in ayu macrophages. Effect of LPS pre-stimulation on the expression of TNF **(A)** and IL-1β **(B)**. Protein levels of pro-inflammatory cytokines were determined by ELISA. Different LPS concentrations were incubated for 6 h followed by 10 μg/ml LPS treatment for an additional 8 h. **(C)** LPS treatment induced the down-regulation of PaSRB2a mRNA. Macrophage cultures were treated with 1 μg/ml LPS. **(D)** PaSRB2a protein levels at 16 h after LPS treatment. Representative blots of three independent experiments are shown. **(E)** Over-expression of PaSRB2a by lentiviral vector in LPS-treated macrophages. Protein levels of TNF **(F)** and IL-1β **(G)** in PaSRB2a over-expressed macrophages after LPS treatment. Each bar represents the mean ± SE *n* = 5. Data are representative of two **(A–C, E–G)** and three **(D)** independent experiments. *P < 0.05, **P < 0.01, ***P < 0.001.

It has long been known that sub-threshold stimulatory concentrations of LPS for 6 h result in the enhancement of pro-inflammatory responses after secondary LPS stimulation in mammals (44). We investigated the effect of secondary LPS stimulation on sub-threshold LPS pre-stimulated ayu macrophages. In 10 μg/ml LPS-stimulated ayu macrophages, the protein levels of TNF and IL-1β were down-regulated at 10, 100, and 1,000 μg/ml LPS pre-stimulated macrophages compared to those without LPS pre-stimulation (Figures 8A,B). We then investigated whether PaSRB2a mediated the down-regulation of pro-inflammatory responses in LPS pre-stimulated macrophages. First, we found that PaSRB2a was down-regulated in LPS pre-stimulated macrophages (Figures 8C,D). We delivered lentivirus to ayu macrophages to increase PaSRB2a expression (Figure 8E). PaSRB2a lentivirus restored the induction of pro-inflammatory responses by LPS in LPS pre-stimulated macrophages (Figures 8F,G). These data demonstrate that sub-threshold stimulatory concentrations of LPS result in the down-regulation of pro-inflammatory responses after secondary LPS stimulation via reducing PaSRB2a expression in ayu.

### Macrophages mediate the effect of paSRB2a on pro-inflammatory responses

**Figure 9:**
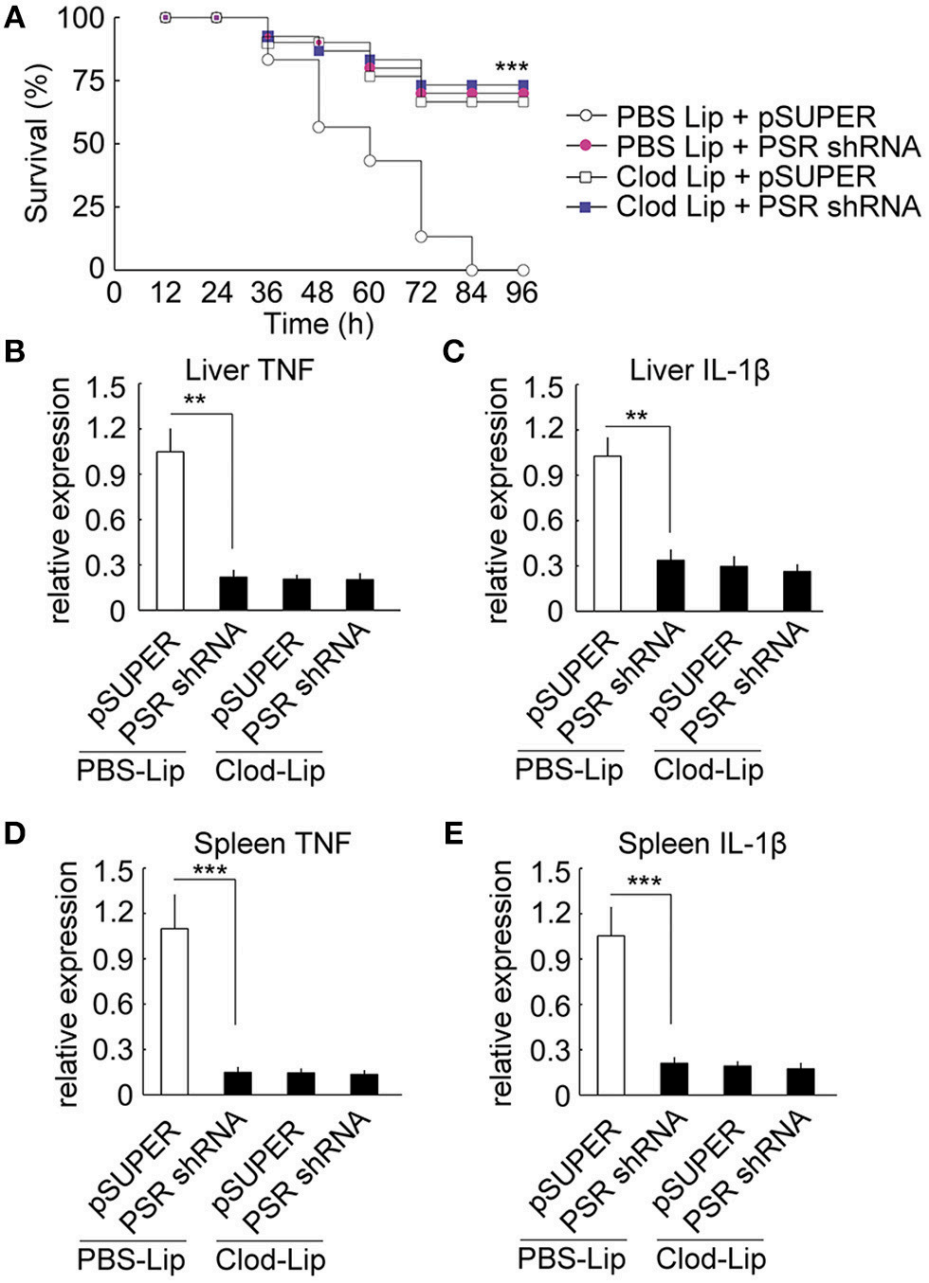
Macrophages mediated PaSRB2a inducement of the pro-inflammatory effect of LPS *in vivo*. **(A)** Survival in ayu treated with pSUPER-PaSRB2a after macrophage depletion. Following macrophage depletion, ayu were treated with LPS after lentivirus delivery. *n* = 30 per group. RT-qPCR analysis of TNF **(B)** and IL-1β **(C)** expression in ayu liver. RT-qPCR analysis of TNF **(D)** and IL-1β **(E)** expression in ayu spleen. Cytokine genes were normalized to 18S rRNA. Each bar represents the mean ± SE *n* = 5. Data are representative of two **(A)** and three **(B–E)** independent experiments. **P < 0.01, ***P < 0.001. PBS Lip: PBS liposome, Clod Lip: clodronate liposome, PSR shRNA: PaSRB2a shRNA.

For the depletion of macrophage, we employed CD115 antibodies to identify ayu monocytes/macrophages in blood by flow cytometry and RT-qPCR. In fish injected with clodronate-liposomes alone, the number of CD115^+^ monocytes/macrophages was significantly decreased in blood compared with that in PBS-liposomes treated ayu (Supplemental Figure 6A). Furthermore, the mRNA expression of CD115^+^ was also down-regulation in blood of cllodronateliposomes treated ayu (Supplemental Figure 6B). Then we employed macrophage-depleted ayu to investigate the effect of PaSRB2a on pro-inflammatory responses in LPS-treated ayu. PaSRB2a shRNA treatment resulted in up-regulation of survival rate in PBS-liposomes treated ayu (Figure 9A), while the survival rate was not altered in the PaSRB2a shRNA-treated group compared with the pSUPER control in macrophage-depleted fish (Figure 9A). Furthermore, PaSRB2a shRNA treatment did not change TNF and IL-1β expression in the liver of macrophage-depleted fish (Figures 9B,C). In the spleen, PaSRB2a shRNA treatment did not change TNF and IL-1β expression in macrophage-depleted fish (Figures 9D,E). These data suggest that macrophages play an important role in PaSRB2a-mediated pro-inflammatory responses to LPS *in vivo*.

### PaSRB2a also mediates the pro-inflammatory response to LPS derived from various bacteria

**Figure 10:**
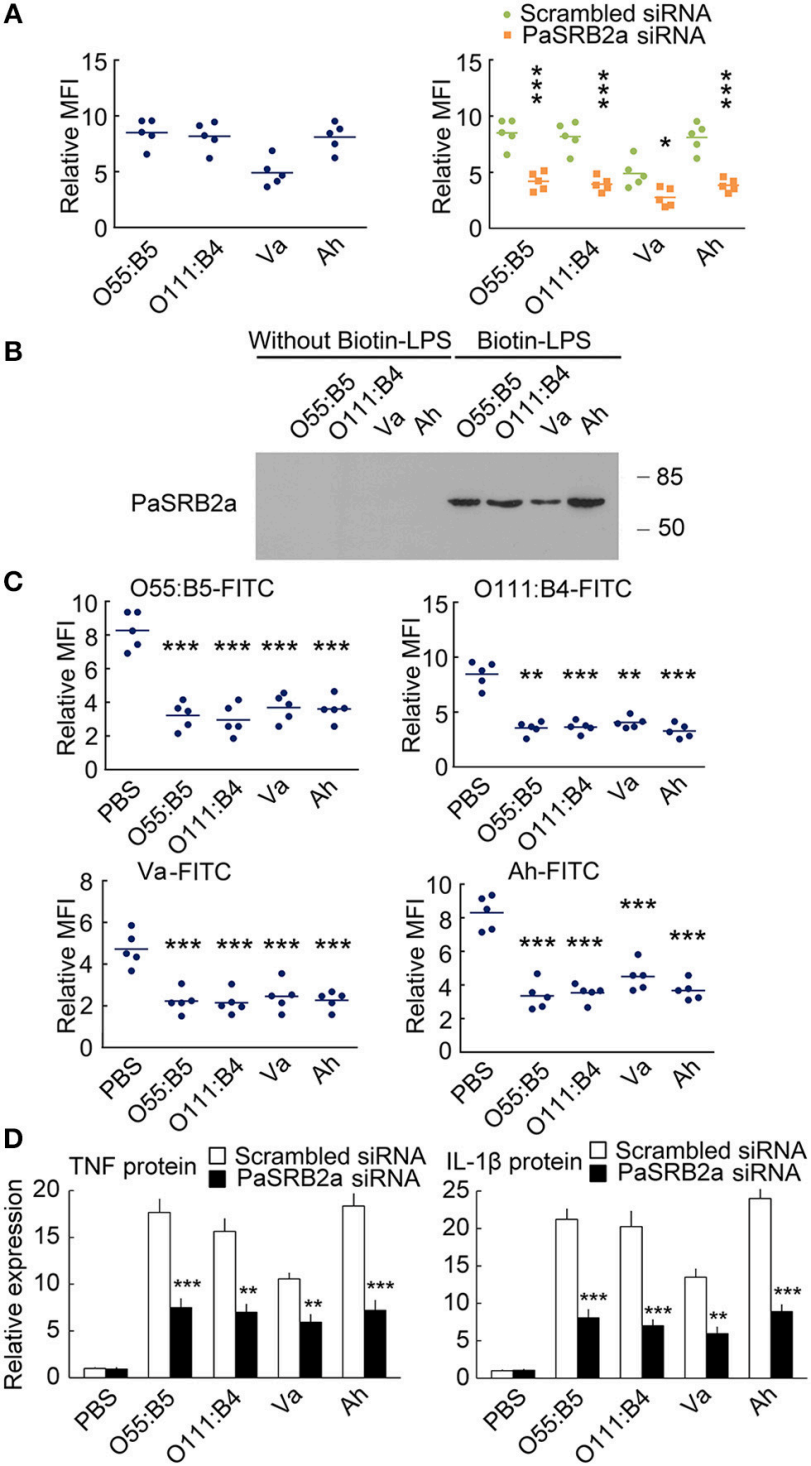
PaSRB2a mediated the inflammatory effect of LPS derived from various bacteria. **(A)** FITC fluorescence was detected in ayu macrophages after treatment with LPS from *E. coli* O55:B5, *E. coli* O111:B4, *V. anguillarum*, or *A. hydrophila*. **(B)** Physical interaction between PaSRB2a and LPS from various bacteria. Representative blots of three independent experiments are shown. **(C)** LPS-FITC was internalized by ayu macrophages after treatment with LPS from various bacteria. **(D)** Protein levels of TNF and IL-1β in ayu macrophages after treatment with LPS from various bacteria. *n* = 5. Data are representative of two **(A, C,D)** and three **(B)** independent experiments. *P < 0.05, **P < 0.01, ***P < 0.001.

We further compare the internalization of LPS from *Escherichia coli* O55:B5, *E. coli* O111:B4, *Vibrio anguillarum*, and *Aeromonas hydrophila* (O55:B5-LPS, O111:B4-LPS, Va-LPS, and Ah-LPS). All four types of LPS were observed to enter ayu macrophages 4 h after treatment (Figure 10A). PaSRB2a siRNA treatment reduced internalization of all 4 types of LPS (Figure 10A). LPS precipitation assay illustrated that PaSRB2a could interact with all types of LPS (Figure 10B). O55:B5-LPS, O111:B4-LPS, Va-LPS, and Ah-LPS antagonized the internalization of LPS from other bacteria, respectively (Figure 10C), suggesting that LPS from different bacteria may employ similar pathways in ayu macrophages. Furthermore, PaSRB2a siRNA treatment reduced TNF and IL-1β expression in ayu macrophages treated with O55:B5-LPS, O111:B4-LPS, Va-LPS, or Ah-LPS, respectively (Figure 10D). These data suggest that PaSRB2a plays an important role in the pro-inflammatory responses of various LPS derived from different bacteria.

### SRB2a-mediated LPS effect on grass carp and spotted green pufferfish

**Figure 11:**
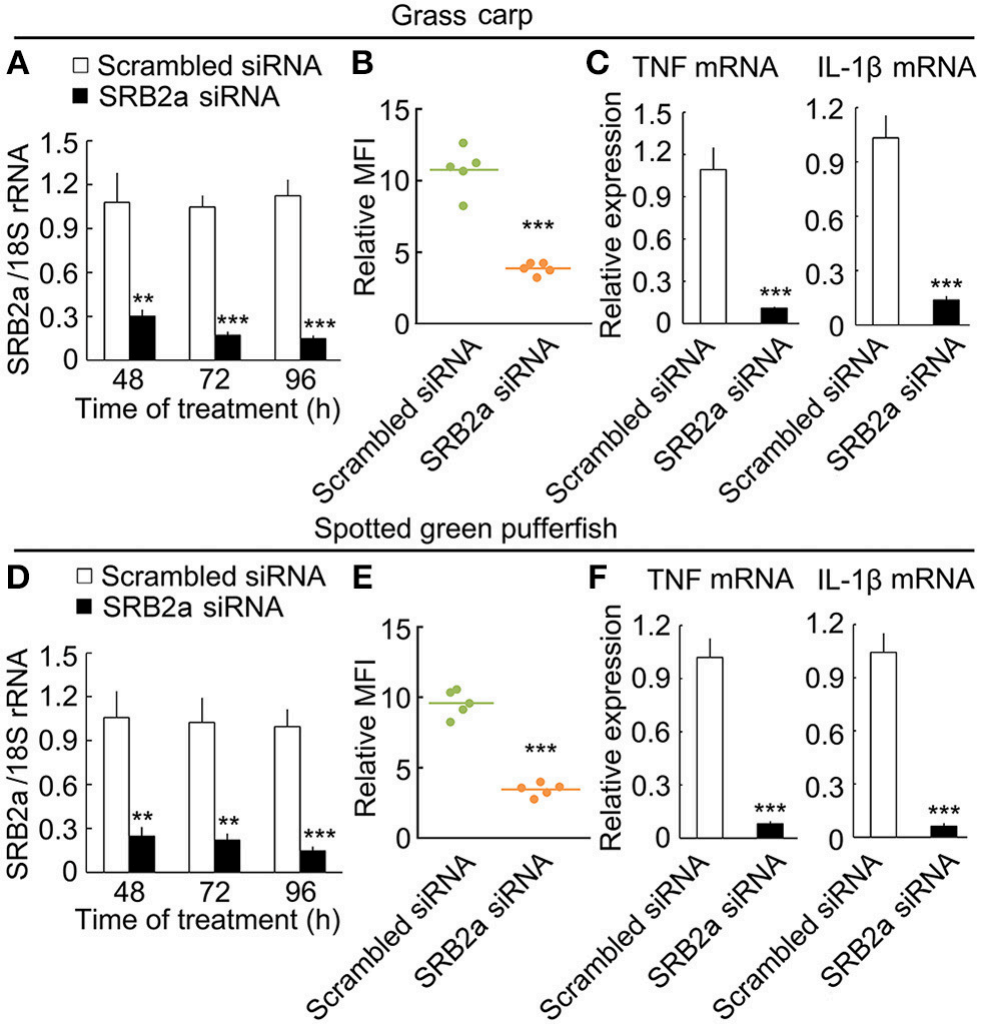
SRB2a mediated LPS internalization and pro-inflammatory effect in the macrophages of grass carp and spotted green pufferfish. **(A)** mRNA levels of SRB2a in macrophages treated with SRB2a siRNA in grass carp. **(B)** LPS detection in macrophages 4 h after 10 μg/ml LPS treatment in grass carp. **(C)** RT-qPCR analysis of TNF and IL-1β expression in grass carp macrophages. **(D)** mRNA levels of SRB2a in macrophages treated with SRB2a siRNA in spotted green pufferfish. **(E)** LPS detection in macrophages 4 h after 10 μg/ml LPS treatment in spotted green pufferfish. **(F)** RT-qPCR analysis of TNF and IL-1β expression in spotted green pufferfish macrophages. *n* = 5. Data are representative of three independent experiments. **P < 0.01, ***P < 0.001.

Since SRB2a also exist in other teleosts, we determined if SRB2a genes in other teleosts mediate LPS internalization and pro-inflammatory responses in macrophages. In grass carp, SRB2a siRNA treatment led to the down-regulation of SRB2a mRNA expression at 48, 72, and 96 h (Figure 11A). SRB2a siRNA treatment reduced LPS internalization in grass carp macrophages (Figure 11B). SRB2a siRNA treatment also decreased TNF and IL-1β mRNA expression in grass carp macrophages (Figure 11C). In spotted green pufferfish, SRB2a siRNA treatment also decreased LPS internalization and pro-inflammatory responses in macrophages (Figures 11D–F). Hence, there may be a common pathway of SRB2a-mediated LPS internalization and pro-inflammatory responses in teleosts.

## Discussion

Although a relatively high concentration of LPS is necessary to activate the immune response of macrophages in teleosts compared to mammals (7), LPS is still an important pathogen-associated molecule involved in the first line of host defense in teleosts (45, 46). Here, we found that LPS internalization was necessary for the pro-inflammatory response in teleost macrophages. SRB2a, a novel gene in teleosts that does not exist in mammals, mediated LPS internalization and pro-inflammatory responses in macrophages. We further identified that SRB2a mediated intracellular activation of NOD1, NOD2, and NF-κB by interacting and internalizing LPS. The C-terminal intracellular domain of SRB2a played an important role in LPS internalization. Moreover, LPS treatment led to the dramatic down-regulation of SRB2a expression in macrophages, which partly explains the LPS tolerance observed in teleosts. Although LPS is an influential pathogen-associated molecule in the teleost immune system (47), this is the first study to characterize the mechanism of LPS internalization to activate pro-inflammatory responses in teleost macrophages.

TLR2 is also a candidate for the LPS signaling receptor in mammals (17, 18). Although several TLR2 genes have been cloned and characterized in teleosts (8, 48), prior to our study it was unclear whether teleost TLR2 is the receptor for LPS. It has been found that two TLR2 genes existed in several fish such as, common carp, large yellow croaker, and rainbow trout (49). Furthermore, the expression of TLR2a is relatively higher in macrophages of common carp compared with TLR2b, suggesting that TLR2a and TLR2b in teleost may play different roles in immune system (49). In ayu, we also found two TLR2 genes, PaTLR2a, and PaTLR2b. PaTLR2a is expressed in ayu macrophages, while PaTLR2b mRNA could not be detected. We also did not find PaTLR2b expression in the transcriptome of ayu macrophages (33). Since PaTLR2b is not expressed in macrophages we further investigated whether PaTLR2a is the LPS receptor in the macrophages of teleosts. We found that PaTLR2a knockdown did not change the effect of LPS on teleost macrophages, suggesting that PaTLR2a is not the teleost LPS receptor. Although further investigation is necessary to demonstrate whether a TLR is the LPS receptor in teleosts, our results support the concept that LPS recognition of teleost macrophages is completely different from that in mammals.

Since the surface receptor for the LPS signaling pathway in teleosts is unclear, it is possible that LPS can enter the cytoplasm to directly activate pro-inflammatory responses in teleosts. In mammals, when the LPS receptor TLR4 in cell surface is knocked out, even 100 μg/ml LPS cannot activate macrophages in mammals (17), suggesting that extracellular LPS itself does not enter macrophages to activate pro-inflammatory responses. In fact, intracellular LPS in mammals is recognized by the outer membrane of gram-negative bacteria-mediated delivery in macrophages (29). In teleost macrophages, 10 μg/ml LPS activated the production of pro-inflammatory cytokines, suggesting that LPS can enter the cytoplasm of teleost macrophages via by a surface receptor.

Mammalian macrophages express SRB, which does not participate in LPS-induced pro-inflammatory responses in mammalian macrophages (17, 50). In teleosts, a scavenger receptor in teleosts has been found to negatively regulate NF-κB activation (51). Here, we found that SRB but not SRA participates in LPS internalization. There are two SRB2 genes in teleosts: SRB2a, which is a novel gene not found in mammals, while SRB2b is the homolog of mammalian SRB2. According to previous studies, redundant genes may regulate the crucial functions of the teleost immune system (36, 52–54). Here, we found that LPS treatment of macrophage culture led to LPS internalization and a pro-inflammatory response mediated by SRB2a. SRB2b knockdown did not affect LPS internalization, and SRB2a knockdown reduced LPS internalization by 55% in teleost macrophages, suggesting that SRB2a is the main receptor in teleost macrophages that mediate LPS internalization. Hence, we have determined that the novel surface receptor SRB2a mediates the pro-inflammatory response in LPS treated teleost macrophages, which differs from CD14- and TLR-mediated LPS responses in mammals. Although mammalian SRB2 in macrophages does not participate in LPS-induced pro-inflammatory responses, SRB2 does indeed mediates bacterial adhesion and uptake in mammalian macrophages (55). Moreover, SRB2 also participates in cytokine production following bacterial infection in mammals (56). These results suggest that mammalian SRB2 also recognize bacterial components to mediate pro-inflammatory response in macrophages, but the LPS may not play a crucial role in the SRB2 effect on mammalian macrophages. Since teleosts lack the LPS recognition signaling proteins, CD14 and MD2, it may be a complement signaling pathway through which teleost SRB2a interacts with LPS. These results also illustrate that the signaling pathways of LPS recognition vary in vertebrates. Further investigation will focus on how teleosts develop different LPS signaling pathway in response to their water environment.

We further investigated the downstream signaling of SRB2a in teleost macrophages. We first found that the C-terminal intracellular domain but not the extracellular domain of SRB2a mediated LPS internalization. Teleost SRB2a transfection alone could not activate NF-κB in HEK293T cells, while the mammalian TLR4 surface receptor complex can activate NF-κB in HEK293T cells (57, 58). These results suggest that PaSRB2a is important for LPS signaling in teleost macrophages, but it may not be the receptor that directly activates the pro-inflammatory responses to LPS. Moreover, we found two important NOD-like receptors expressed in teleost macrophages, PaNOD1 and PaNOD2 (33). PaNOD1 and PaNOD2 knockdown led to stronger down-regulation of the pro-inflammatory responses in teleost macrophages. These data suggest that NOD1 and NOD2 are the main intracellular receptors of LPS in teleost macrophages following LPS internalization mediated by SRB2a.

Since SRB2a downstream signaling in teleosts differs from TLR4 in mammals, we further investigated whether the unique LPS recognition mechanism of SRB2a affects LPS priming and tolerance. Although LPS tolerance is observed after LPS pre-stimulation for 24 h (59), LPS pre-stimulation with sub-threshold concentrations for 6 h increase the pro-inflammatory responses in mammalian macrophages (44). Here, we found that LPS pre-stimulation with sub-threshold concentrations for 6 h reduced the pro-inflammatory responses in teleost macrophages. Moreover, PaSRB2a was down-regulated in teleost macrophages treated with a sub-threshold LPS concentration. Over expression of PaSRB2a restored the secondary LPS treatment-induced pro-inflammatory responses. Hence, the down-regulation of SRB2a expression after LPS induction suggests a negative feedback regulation of LPS in teleost macrophages. In mammalian macrophages, the expression of LPS receptor TLR4 is not down-regulated after LPS treatment (60). Firstly, the TLR4 surface receptor complex in mammals can activate NF-κB, but SRB2a in teleosts cannot. Secondly, SRB2a in teleosts is down-regulated after LPS treatment, but TLR4 in mammals is not. These results may partly explain the LPS-tolerant mechanism in teleosts. Our results also support the concept that differences in immune responses between teleosts and mammals contribute to the understanding of the evolution of the immune system in vertebrates (61). The interaction of LPS with its receptor is critical for immune responses after bacterial infections, and its dysregulation is thought to promote aberrant cytokine production during bacterial sepsis (62), when LPS release during infection is enhanced when bacterial biomass increases (63). Hence, LPS detection is a crucial process when bacterial numbers increases *in vivo* during infection. Pre-treatment with low doses of LPS leads to increased pro-inflammatory responses in mammalian macrophages in the early stages of infection, while the decreased pro-inflammatory responses seen in teleost macrophages may contribute to their LPS tolerance. In mammals, knockout of the LPS receptor TLR4 confers resistance to bacteria induced sepsis (64), suggesting that dramatic LPS-induced pro-inflammatory responses are detrimental in sepsis. LPS tolerance in teleosts may therefore be beneficial to severe infection. Further investigation is necessary to illustrate the differential mechanisms underlying the recognition of pathogen-associated molecular patterns between teleosts and mammals.

LPS, a major component of the outer membrane of gram-negative bacteria, is important to the host defense when vertebrates are infected with bacteria. Although teleosts are less sensitive to LPS compared with mammals, LPS is lethal at high doses (65). In both teleosts and mammals, LPS is thought to be an important bacterial component, inducing cytokine expression and death (66). Here, we found that the survival rate was up-regulated and the expression of pro-inflammatory cytokines was down-regulated in LPS-treated ayu that had been administered with lentivirus containing specific small hairpin RNAs, targeting teleost SRB2a. Furthermore, SRB2a shRNA treatment did not change survival rates or the expression of pro-inflammatory cytokines in macrophage-depleted ayu. This further suggests that teleost SRB2a in macrophages regulates pro-inflammatory responses *in vivo*.

**Figure 12:**
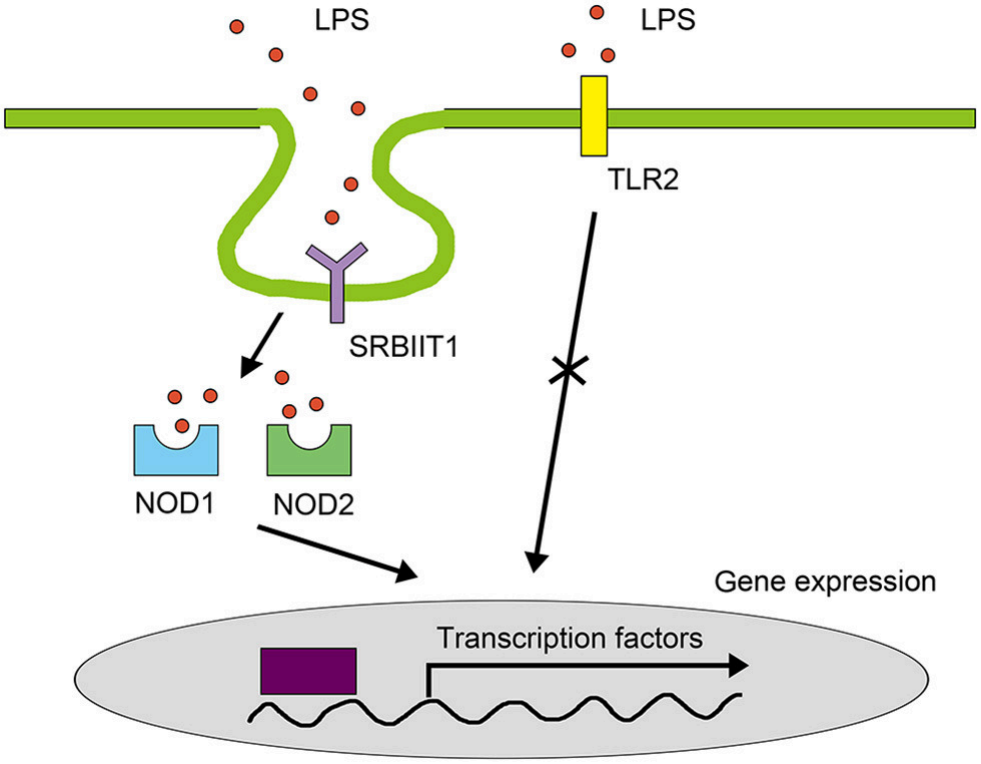
LPS signaling pathway in teleost macrophages. In teleosts, SRB2a mediates LPS internalization for interaction with NOD1 and NOD2 to initiate NF-κB in macrophages. TLRs do not mediate LPS signals in the pro-inflammatory response of teleost macrophages.

In summary, our study characterized a novel pathway for LPS signaling in the macrophages of vertebrates (Figure 12). Although we do not exclude the existence of other LPS signals in teleost immune cells, to our knowledge, this is the first study to reveal an LPS receptor on the cell surface that mediates pro-inflammatory responses in teleosts. This study illustrates that the LPS signaling in teleosts are different from that in mammals, contributing to our understanding of the evolution of pathogen recognition of vertebrates.

## Data availability statement

The sequences presented in this article have been submitted to GenBank (http://www.ncbi.nlm.nih.gov/genbank/) under the accession numbers MG674831 (PaTLR2), MH699855 (PaSRB2a), JP736791 (PaSRB2b), MG674829 (PaNOD1), and MG674830 (PaNOD2).

## Author contributions

X-JL, Y-JN, HL, and LN performed the experiments and data analysis. X-JL and JC designed the *in vivo* and *in vitro* experiments. X-JL and JC wrote the manuscript and JC directed the study.

### Conflict of interest statement

The authors declare that the research was conducted in the absence of any commercial or financial relationships that could be construed as a potential conflict of interest.
