# Supplementary material for: A Novel Lipopolysaccharide Recognition Mechanism Mediated by Internalization in Teleost Macrophages
Source: Front Immunol. 2018 Nov 27;9:2758. doi: 10.3389/fimmu.2018.02758 (PMC6277787; doi:10.3389/fimmu.2018.02758)
Supplement: Supplementary file 1 [file Data_Sheet_1.PDF]

Supplementary Material

A Novel Lipopolysaccharide Recognition Mechanism Mediated by Internalization in Teleost Macrophages

Xin-Jiang Lu[1,2,]*, Ying-Jun Ning[1], He Liu[1], Li Nie[1,2], Jiong Chen[1,2,]*

***Correspondence:**
Xin-Jiang Lu, lxj711043@163.com; Jiong Chen, jchen1975@163.com

1 Supplementary Figures

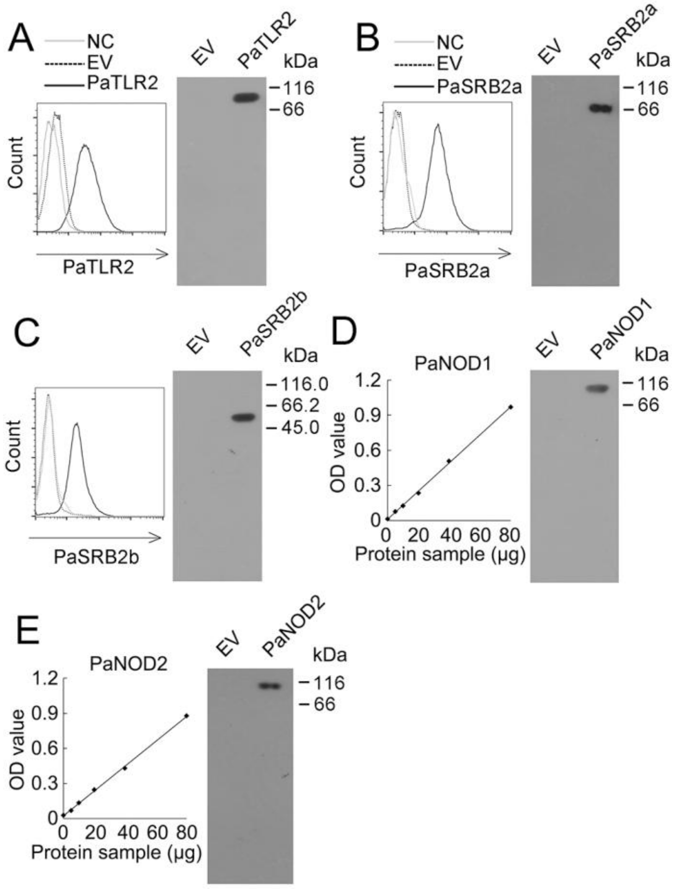

Supplementary Figure 1. Validation of the specificity of antibodies used in this study. **(A)** Flow cytometry and western blot analysis of HEK-293T cells expressing empty vector or PaTLR2. **(B)** Flow cytometry and western blot analysis of HEK-293T cells expressing empty vector or PaSRB2a. **(C)** Flow cytometry and western blot analysis of HEK-293T cells expressing empty vector or

PaSRB2b. **(D)** ELISA and western blot analysis of HEK-293T cells expressing empty vector or PaNOD1. **(E)** ELISA and western blot analysis of HEK-293T cells expressing empty vector or PaNOD2. EV: empty vector. Representative blots of three independent experiments are shown. Each bar represents the mean ± SE. Data are representative of three independent experiments.

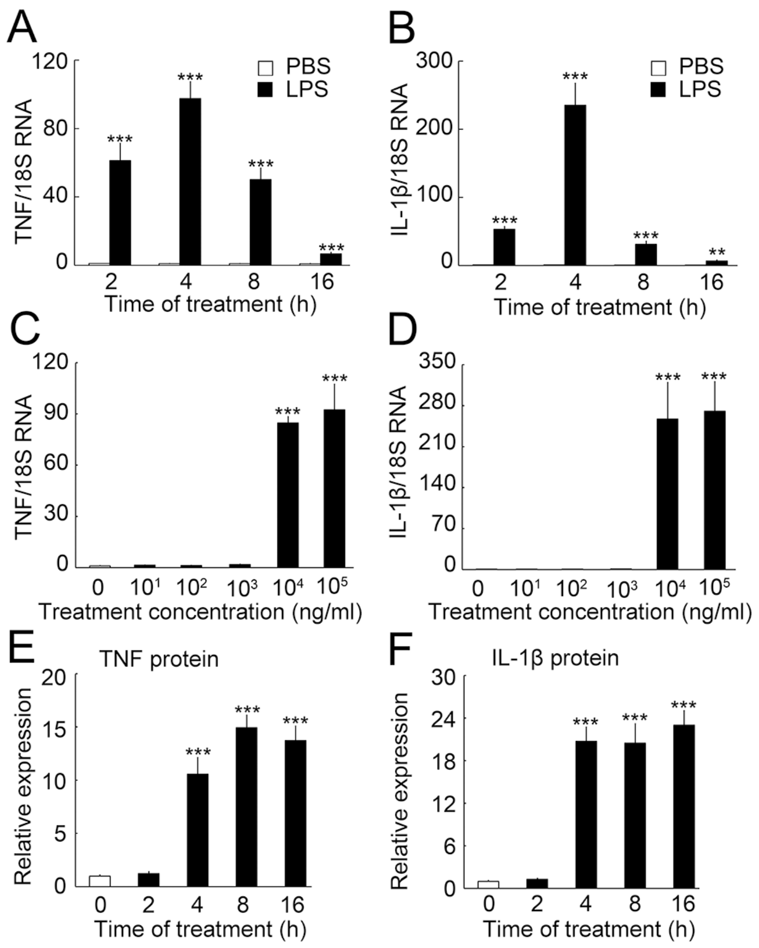

Supplementary Figure 2. LPS induces the expression of pro-inflammatory cytokines TNF and IL-1β in macrophages from ayu. The mRNA levels of the pro-inflammatory cytokines TNF **(A)** and IL-1β **(B)** were determined by RT-qPCR. Macrophages were stimulated for 2, 4, 8, and 16 h with 10 μg/mL LPS (serotypes O55:B5). Gene expression was normalized against 18S RNA and is shown as relative to the mean of non-stimulated cells. The mRNA levels of TNF **(C)** and IL-1β **(D)** were determined by RT-qPCR. Macrophages were stimulated for 4 h with 0, 10^1, 10^2, 10^3, 10^4, and 10^5 ng/mL LPS. The protein levels of TNF **(E)** and IL-1β **(F)** were determined by ELISA. Each bar represents the mean ± SE. n = 5. Data are representative of three independent experiments. $^{**}P < 0.01$, $^{***}P < 0.001$.

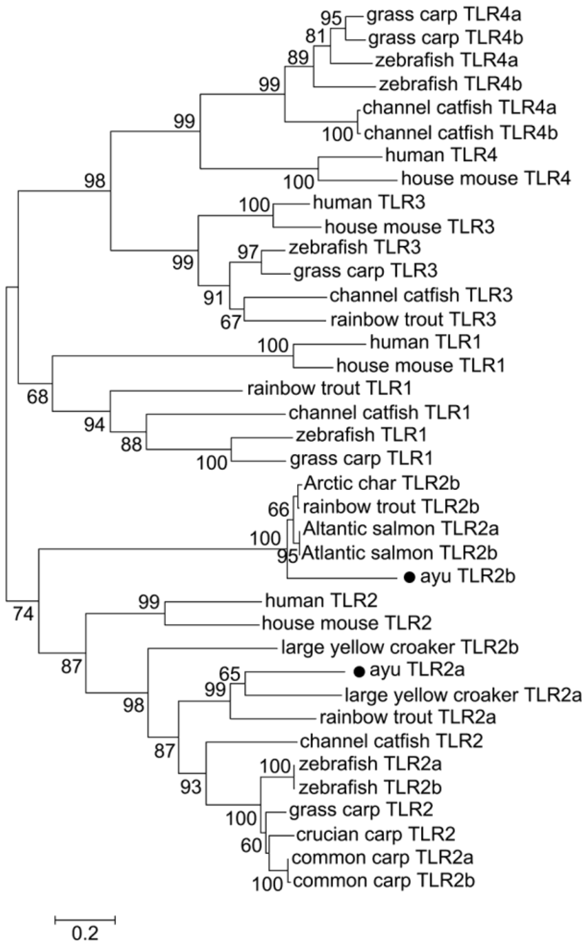

Supplementary Figure 3. Phylogenetic (neighbor-joining) analysis of amino acid sequences of TLRs using MEGA5.0. Node values represent the percentage bootstrap confidence derived from 1000 replicates. The accession numbers of TLRs are listed in Table 3.

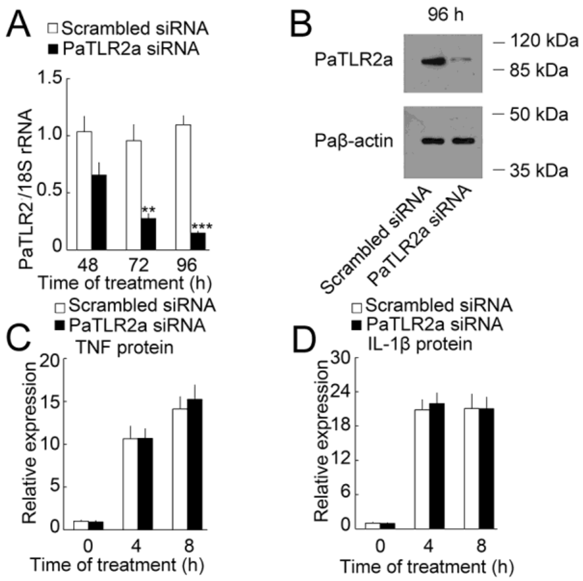

Supplemental Figure 4. PaTLR2a effect on TNF and IL-1β production in LPS treated ayu macrophages. **(A)** mRNA expression of PaTLR2a in macrophages treated with PaTLR2a siRNA. **(B)** Protein level of PaTLR2a in macrophages treated with PaTLR2a siRNA. Representative blots of three independent experiments are shown. The protein levels of TNF **(C)** and IL-1β (D) were determined by ELISA. Macrophages were stimulated for 4 and 8 h with 10 μg/mL LPS. Each bar represents the mean ± SE. n = 5. Data are representative of three (A, C-D) and two (B) independent experiments. $**P < 0.01$, $***P < 0.001$.

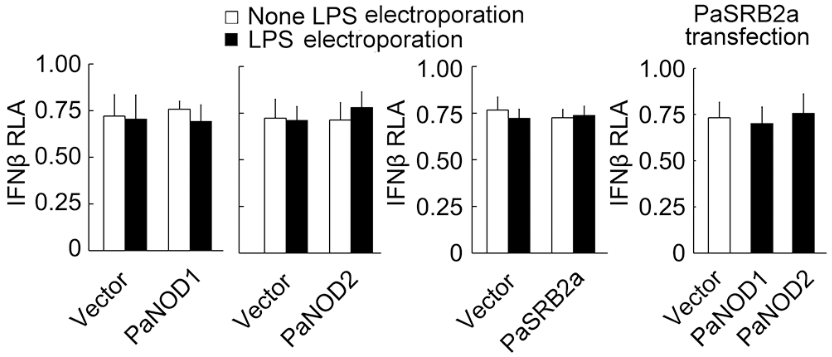

Supplementary Figure 5. LPS activated NF-κB via PaNOD1, PaNOD2, and PaSRB2a. **(E)** No IFNβ activation was observed in LPS-treated HEK293T cells after transfection with PaSRB2a, PaNOD1, or PaNOD2. Representative blots of three independent experiments are shown. Each bar represents the mean ± SE. n = 5. Data are representative of at least three independent experiments. $^{**}P < 0.01$, $^{***}P < 0.001$.

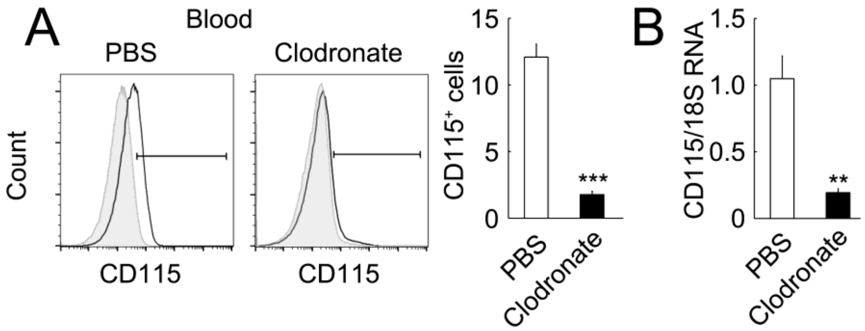

Supplementary Figure 6. Effect of clodronate-liposome injection on ayu monocytes/macrophages. (A) Ayu were injected ip with PBS- or clodronate-liposomes. Mononuclear cells from blood were labeled with CD115 for flow cytometry analysis. Histograms show CD115 expression in the cells of ayu blood. (B) Percentage and mRNA expression of CD115$^+$ cells in mononuclear cells from blood of ayu treated with PBS- or clodronate-liposomes. Each bar represents the mean ± SE. n = 5. Data are representative of at least three independent experiments. $^{**}P < 0.01$, $^{***}P < 0.001$.
